# Correction: The NOD-Like Receptor Signalling Pathway in *Helicobacter pylori* Infection and Related Gastric Cancer: A Case-Control Study and Gene Expression Analyses

**DOI:** 10.1371/journal.pone.0117870

**Published:** 2015-01-30

**Authors:** The PLOS ONE Staff

## Notice of Republication

This article was republished on October 28, 2014 to include information missing from the “*Helicobacter Pylori* Infection Results in Up-regulation of *NFKB1* with Simultaneous Marked down-Regulation of *NLRP12* and *NLRX1*” subsection of the Results. Please download this article again to view the correct version. The originally published, uncorrected article and the republished, corrected article are provided here for reference.

## Supporting Information

S1 File Originally published, uncorrected article. (PDF)

S2 File Republished corrected article. (PDF)
