# Supplementary figures and images for: The NOD-Like Receptor Signalling Pathway in Helicobacter pylori Infection and Related Gastric Cancer: A Case-Control Study and Gene Expression Analyses
Source: PLoS One. 2014 Jun 5;9(6):e98899. doi: 10.1371/journal.pone.0098899 (PMC4047072; doi:10.1371/journal.pone.0098899)

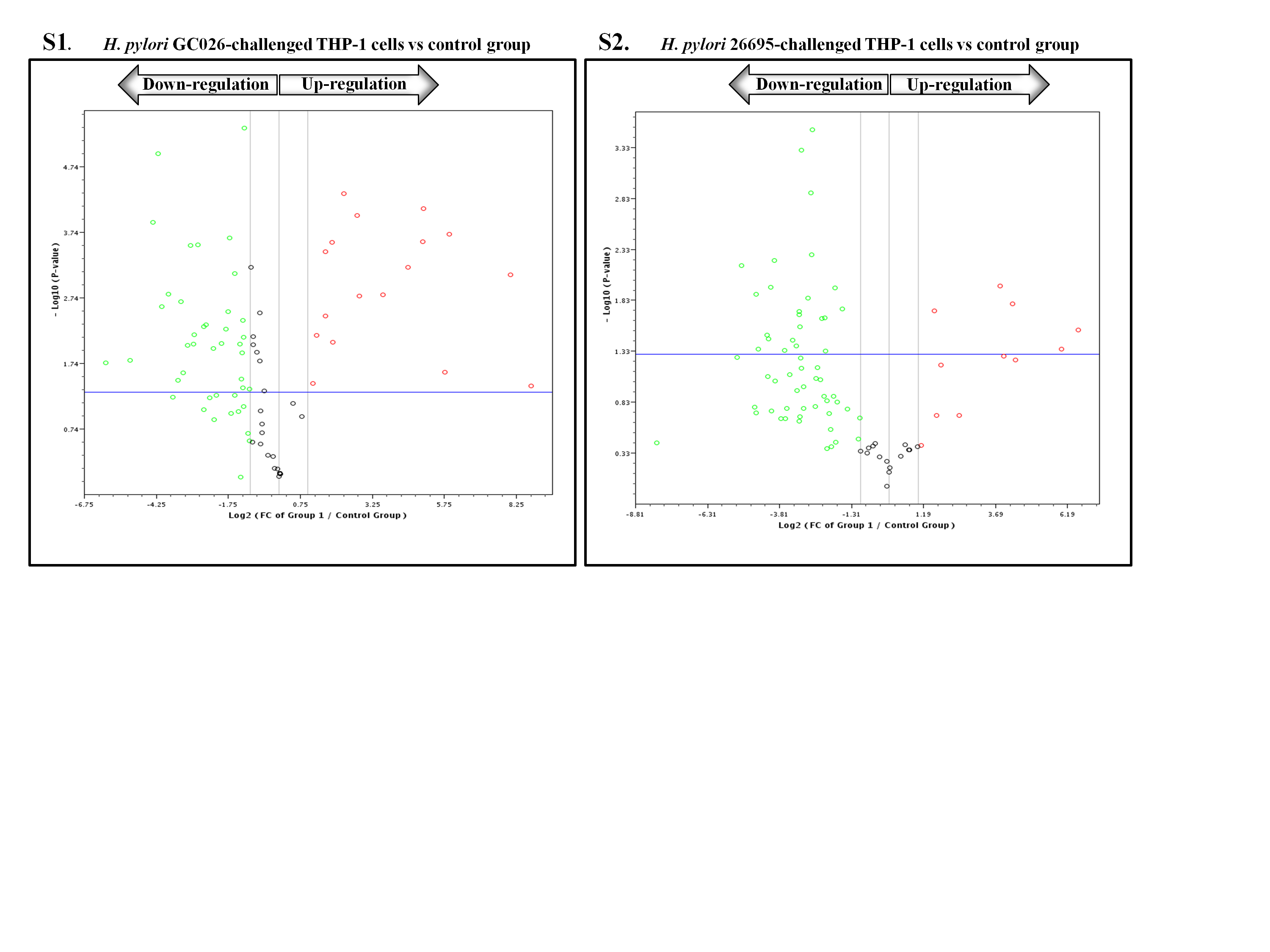

Supplement: Figures S1 — This file contains Figures S1 and S2. Helicobacter pylori influences the expression of several molecules involved in the NOD-like receptor signalling pathway. THP-1 cells were challenged with two H. pylori strains (GC026 and 26695). Total RNA was extracted from cells after 6 hours infection. Gene expression was detected by quantitative RT-PCR in triplicates. S1) Gene expression of 84 molecules involved in the NOD-like receptor (NLR) signalling pathway in H. pylori GC026-challenged THP-1 cells. S2) Gene expression of 84 molecules involved in the NLR signalling pathway in H. pylori 26695-challenged THP-1 cells. The x-axis plots the log2 of the fold-differences, while the y-axis plots their p-values based on a student’s t-test of the replicate raw Ct data. The red and green circles outside the two vertical lines indicate fold-differences >2. Circles in the volcano plot above the blue line identify fold-differences showing P-values<0.05. (TIF) [file pone.0098899.s001.tif]
