# Supplementary material for: The NOD-Like Receptor Signalling Pathway in Helicobacter pylori Infection and Related Gastric Cancer: A Case-Control Study and Gene Expression Analyses
Source: PLoS One. 2014 Jun 5;9(6):e98899. doi: 10.1371/journal.pone.0098899 (PMC4047072; doi:10.1371/journal.pone.0098899)
Supplement: Tables S1 — This contains files Tables S1–S7. Table S1. Genetic polymorphisms in genes involved in the NOD-like receptors signalling pathway included in the current study. Table S2. PCR primer sequences and thermal conditions used for genotyping of NLRP3 and CARD8 polymorphisms in gastric cancer patients and functional dyspepsia controls. Table S3. Clinical characteristics of gastric cancer patients and functional dyspepsia controls. Table S4. Association between the NLRP3 42 bp-VNTR polymorphism and risk of gastric cancer in ethnic Chinese individuals (bivariate statistical analysis). Table S5. Association between CARD8-NLRP12, NLRP3, NLRX1-CASP1 and ASC haplotypes and gastric cancer in an ethnic Chinese population. Table S6. Association between polymorphisms involved in the NOD-like receptors signalling pathway and risk of Helicobacter pylori infection in ethnic Chinese individuals (bivariate statistical analysis). Table S7. Effect of Helicobacter pylori infection on the expression of genes involved in the NOD-like receptors signalling pathway. (DOC) [file pone.0098899.s003.doc]

**Table S1.** Genetic polymorphisms in genes involved in the NOD-like receptors signalling pathway included in the current study.

| **Gene** | **Polymorphisms** |
| --- | --- |
| *NLRP3* | rs10754558, rs10925026, rs12079994, rs1539019, rs3806265, rs4612666, rs4925650, rs104895409^*^, rs104895411^*^, rs59878320^*^, rs6677787^*^, rs71642413^*^, 42 bp-VNTR |
| *NLRP12* | rs104895565, rs199475867, rs199475868^*^, rs199475869^*^, rs2866112, rs35768562^*^, rs4419163, rs4539722 |
| *NLRX1* | rs10790286, rs142957206^*^, rs199475871^*^, rs199476053^*^ |
| *ASC* | rs116026792^*^, rs11648861^*^, rs151056688, rs200371118^*^, rs79464842^*^, rs8056505 |
| *CASP1* | rs151040610^*^, rs183446148^*^, rs199935430^*^, rs202211771^*^, rs2282659, rs501192, rs530537, rs56039250^*^, rs61751523, rs61755276^*^ |
| *CARD8* | rs10405717, rs11670259, rs11672725, rs12984929, rs16981829, rs1966625, rs204321*, rs4802445, rs4802449, rs6509368 |

*non-polymorphic in this ethnic Chinese population.

**Table S2. PCR primer sequences and thermal conditions used for genotyping of *NLRP3* and *CARD8* polymorphisms in gastric cancer patients and functional dyspepsia controls.**

| **Polymorphism** | **Primer** | **Sequence** | **Tm ^*^** | **Tm ^†^** | **Tm^‡^** | **Cycles** |
| --- | --- | --- | --- | --- | --- | --- |
| *NLRP3* 42bp-VNTR | F | 5' CTGACCTCCCAATGTGCCTT 3' | 95° C for 30 s | 59°C for 30 s | 72°C for 1 min | 35 |
|  | R | 5' GCTTCAGTCCCACACACAGA 3' |  |  |  |  |
| *NLRP3* rs10754558^£^ | F1 | 5' CAACAATGACAGCATCGGGTGTTGTTG 3' | S2: 95°C for 15 s | S2: 69°C for 20 s | S2: 72°C for 20 s | 10 |
|  | F2 | 5' GAACAATGACAGCATCGGGTGTTGTTC 3' | S3: 95°C for 15 s | S3: 60°C for 30 s | S3: 72°C for 10 s | 40 |
|  | R | 5' AATAGAAAGATAGCGGGAATGA 3' |  |  |  |  |
| *CARD8* rs12984929^£^ | F1 | 5’CAACCCTTCTGTCTCCATAGGTTG 3’ | S2: 95°C for 15 s | S2: 69°C for 20 s | S2: 72°C for 20 s | 10 |
|  | F2 | 5’AAACCCTTCTGTCTCCATAGGTTT 3’ | S3: 95°C for 15 s | S3: 60°C for 30 s | S3: 72°C for 10 s | 40 |
|  | R | 5' TCGGTCCATAACAACTCC 3' |  |  |  |  |
| *CARD8* rs2043211^£^ | F1 | 5' TTCCGCACTCAGGAACAGCACGGAA 3' | S2: 95°C for 15 s | S2: 69°C for 20 s | S2: 72°C for 20 s | 10 |
|  | F2 | 5' ATCCGCACTCAGGAACAGCACGGAT 3' | S3: 95°C for 15 s | S3: 60°C for 30 s | S3: 72°C for 10 s | 40 |
|  | R | 5' TGTTGTTGTTTTCCAGGGTA 3' |  |  |  |  |
| *CARD8* rs6509368^£^ | F1 | 5' CGATATATTGATGTTAGCATATCG 3' | S2: 95°C for 15 s | S2: 64°C for 20 s | S2: 72°C for 20 s | 10 |
|  | F2 | 5' TGATATATTGATGTTAGCATATA 3' | S3: 95°C for 15 s | S3: 55°C for 30 s | S3: 72°C for 10 s | 40 |
|  | R | 5' CACAAAAGACAAGGGAAGAA 3' |  |  |  |  |

PCR, polymerase chain reaction; Tm, temperature; VNTR, variable number of tandem repeat; F, forward; R, reverse; S2, stage 2; S3, stage 3.

^*^ Denaturation temperature.

**^†^**  Annealing temperature.

**^‡^** Extension temperature.

^£^ Stage 1 is 95°C for 10 min.

**Table S3. Clinical characteristics of gastric cancer patients and functional dyspepsia controls.**

| **Characteristic** | **GC cases**  **(N=87)** | **FD controls**  **(N=223)** |
| --- | --- | --- |
| Male/Female N | 55/32 | 99/124 |
| *H. pylori* positive N (%) | 73 (83.9) | 140 (62.8) |
| Median age ± SD (years) | 65.3 ± 13.2 | 54.3 ± 12.8 |

GC, gastric cancer; FD, functional dyspepsia; N, total number; SD, standard deviation.

**Table S4.** Association between the *NLRP3* 42bp-VNTR polymorphism and risk of gastric cancer in ethnic Chinese individuals (bivariate statistical analysis).

| **Genotype analysis** | **OR** | **95% CI** | ***P*-value^*^** |
| --- | --- | --- | --- |
| 12/12 vs 12/9 | 0.54 | 0.11-2.64 | 0.7254 |
| 12/12 vs 12/7 | 0.55 | 0.27-1.13 | 0.1275 |
| 12/12 vs 12/6 | 0.91 | 0.44-1.88 | 0.8553 |
| 12/12 vs 9/9 | N/A | N/A | N/A |
| 12/12 vs 9/7 | 4.33 | 0.38-48.89 | 0.2438 |
| 12/12 vs 9/6 | 0.43 | 0.02-9.14 | 1 |
| 12/12 vs 7/7 | 0.54 | 0.06-4.97 | 1 |
| 12/12 vs 7/6 | 0.93 | 0.23-3.74 | 1 |
| 12/12 vs 6/6 | 0.58 | 0.18-1.83 | 0.4352 |
| **Allele analysis** | **OR** | **95% CI** | ***P*-value^*^** |
| 12 vs other repeats | 0.78 | 0.53-1.15 | 0.244 |
| 12 vs 9 | 0.87 | 0.27-2.78 | 1 |
| 12 vs 7 | 0.72 | 0.41-1.25 | 0.2855 |
| 12 vs 6 | 0.82 | 0.49-1.36 | 0.5291 |

OR, odds ratio; CI, confidence intervals; NA, not applicable. ^*^ Fisher’s exact test two-tailed *P*-value.

**Table S5.** Association between *CARD8-NLRP12*, *NLRP3*, *NLRX1-CASP1* and *ASC* haplotypes and gastric cancer in an ethnic Chinese population.

| ***CARD8-NLRP12* haplotypes** | | | | | | |
| --- | --- | --- | --- | --- | --- | --- |
| **rs10405717, rs11670259, rs11672725, rs16981829, rs1966625, rs2043211, rs4802449, rs104895565, rs199475867, rs2866112, rs4419163, rs4539722** | | | | | | |
| **Name** | **Definition** | **GC cases** | **FD controls** | **OR** | **95%CI** | ***P*-value** |
| Hap1 | C C T C G T G DEL T C T A | 0.067 | 0.008 | **5.402** | **1.58-18.51** | **0.008** |
| Hap2 | C T T T G A A DEL T C T A | 0.065 | 0.038 | 1.271 | 0.52-3.08 | 0.65 |
| Hap3 | C C C T A A G DEL T C T A | 0.064 | 0.096 | 0.5145 | 0.23-1.15 | 0.1219 |
| Hap4 | C C C C G T G DEL T C T G | 0.049 | 0.027 | 1.31 | 0.48-3.57 | 0.6099 |
| Hap5 | T C C C G T G DEL T G A G | 0.048 | 0.017 | 2.245 | 0.74-6.83 | 0.1591 |
| Hap6 | C C C C G T G DEL T C A G | 0.040 | 0.017 | 1.964 | 0.63-6.16 | 0.248 |
| Hap7 | C C C T A A G DEL T C A G | 0.039 | 0.026 | 1.25 | 0.44-3.58 | 0.7859 |
| Hap8 | C C C C G T G DEL T G T A | 0.037 | 0.004 | **5.893** | **1.12-31.12** | **0.0485** |
| Hap9 | C T C T G A A DEL T C T A | 0.023 | 0.025 | 0.7143 | 0.21-2.45 | 0.7675 |
| Hap10 | C C C T G A G DEL T C T A | 0.023 | 0.013 | 1.31 | 0.34-5.03 | 0.7322 |
| Hap11 | C T T T G A A DEL T C T G | 0.021 | 0.028 | 0.6548 | 0.19-2.22 | 0.5723 |
| ***NLRP3* haplotypes** | | | | | | |
| **rs10754558, rs10925026, rs12079994, rs1539019, rs3806265, rs4612666, rs4925650** | | | | | | |
| **Name** | **Definition** | **GC cases** | **FD controls** | **OR** | **95%CI** | ***P*-value** |
| Hap12 | G C G T T C A | 0.224 | 0.198 | 0.9525 | 0.57-1.60 | 0.8949 |
| Hap13 | C A G G T C A | 0.084 | 0.105 | 0.656 | 0.33-1.32 | 0.3096 |
| Hap14 | C A A G C T G | 0.065 | 0.071 | 0.7649 | 0.35-1.66 | 0.5699 |
| Hap15 | C C G T C T G | 0.065 | 0.013 | **3.952** | **1.38-11.36** | **0.0133** |
| Hap16 | G A G G T C A | 0.057 | 0.086 | 0.5826 | 0.27-1.28 | 0.1983 |
| Hap17 | C A G G T C G | 0.037 | 0.019 | 1.617 | 0.53-4.94 | 0.3881 |
| Hap18 | C A G G T T A | 0.034 | 0.020 | 1.437 | 0.48-4.28 | 0.5664 |
| Hap19 | G C G T C T G | 0.031 | 0.059 | 0.4145 | 0.15-1.15 | 0.1243 |
| Hap20 | C C A T C T G | 0.030 | 0.015 | 1.796 | 0.52-6.20 | 0.3405 |
| ***NLRX1-CASP1* haplotypes** | | | | | | |
| **rs10790286, rs2282659, rs501192, rs530537, rs61751523** | | | | | | |
| **Name** | **Definition** | **GC cases** | **FD controls** | **OR** | **95%CI** | ***P*-value** |
| Hap21 | C A G A T | 0.246 | 0.209 | **3.156** | **1.23-8.09** | **0.0175** |
| Hap22 | T G G G T | 0.153 | 0.197 | 0.8588 | 0.51-1.44 | 0.6054 |
| Hap23 | C G G G T | 0.132 | 0.068 | **2.253** | **1.22-4.16** | **0.0117** |
| Hap24 | T A G A C | 0.058 | 0.021 | **3.156** | **1.23-8.09** | **0.0175** |
| ***ASC* haplotypes** | | | | | | |
| **rs151056688, rs8056505** | | | | | | |
| **Name** | **Definition** | **GC cases** | **FD controls** | **OR** | **95%CI** | ***P*-value** |
| Hap25 | C C | 0.285 | 0.263 | 1.168 | 0.78-1.74 | 0.4713 |

GC, gastric cancer; FD, functional dyspepsia; OR, odds ratio; CI, confidence intervals.

**Table S6.** Association between polymorphisms involved in the NOD-like receptors signalling pathway and risk of *Helicobacter pylori* infection in ethnic Chinese individuals (bivariate statistical analysis).

| **Gene** | **Polymorphism** | **Nucleotide change** | **HP-positive individuals (2N=416)** | | **HP-negative individuals (2N=192)** | | **OR** | **95% CI** | ***P*-value^*^** |
| --- | --- | --- | --- | --- | --- | --- | --- | --- | --- |
|  |  |  | **X** | **Y** | **X** | **Y** |  |  |  |
| *CARD8* | rs10405717 | C>T | 285 | 125 | 127 | 59 | 0.94 | 0.65-1.37 | 0.7746 |
|  | rs11670259 | C>T | 328 | 82 | 150 | 36 | 1.04 | 0.67-1.61 | 0.9119 |
|  | rs11672725 | C>T | 317 | 89 | 145 | 37 | 1.10 | 0.72-1.69 | 0.7445 |
|  | rs16981829 | T>C | 198 | 208 | 89 | 95 | 0.98 | 0.69-1.40 | 1 |
|  | rs1966625 | G>A | 326 | 82 | 152 | 36 | 1.06 | 0.69-1.64 | 0.8256 |
|  | rs2043211 | A>T | 198 | 212 | 91 | 95 | 1.03 | 0.73-1.45 | 0.9296 |
|  | rs4802449 | G>A | 316 | 94 | 143 | 43 | 0.99 | 0.66-1.49 | 1 |
| *NLRP3* | rs10754558 | C>G | 239 | 169 | 117 | 69 | 1.20 | 0.84-1.71 | 0.3227 |
|  | rs10925026 | A>C | 237 | 173 | 116 | 72 | 1.18 | 0.83-1.68 | 0.4203 |
|  | rs12079994 | G>A | 363 | 47 | 159 | 29 | 0.71 | 0.43-1.17 | 0.1873 |
|  | rs1539019 | G>T | 236 | 172 | 114 | 74 | 1.12 | 0.79-1.60 | 0.5322 |
|  | rs3806265 | T>C | 211 | 199 | 93 | 95 | 0.92 | 0.65-1.30 | 0.6606 |
|  | rs4612666 | C>T | 216 | 192 | 90 | 98 | 0.82 | 0.58-1.15 | 0.2533 |
|  | rs4925650 | G>A | 225 | 181 | 100 | 82 | 0.98 | 0.69-1.39 | 0.9287 |
| *NLRP12* | rs104895565 | A>del | 410 | 4 | 192 | 0 | 4.22 | 0.23-78.84 | 0.3131 |
|  | rs199475867 | T>G | 408 | 6 | 189 | 3 | 1.08 | 0.27-4.36 | 1 |
|  | rs2866112 | C>G | 304 | 110 | 159 | 31 | **1.86** | **1.19-2.89** | **0.0052** |
|  | rs4419163 | T>A | 284 | 120 | 148 | 38 | **1.65** | **1.09-2.49** | **0.0212** |
|  | rs4539722 | A>G | 200 | 212 | 107 | 81 | 0.71 | 0.50-1.01 | 0.0645 |
| *NLRX1* | rs10790286 | T>C | 264 | 150 | 138 | 52 | **1.51** | **1.04-2.20** | **0.033** |
| *ASC* | rs151056688 | C>T | 396 | 0 | 187 | 1 | 0.16 | 0.01-3.89 | 0.3219 |
|  | rs8056505 | T>C | 289 | 125 | 149 | 43 | **1.50** | **1.01-2.23** | **0.0511** |
| *CASP1* | rs2282659 | A>G | 307 | 105 | 130 | 60 | 0.74 | 0.51-1.08 | 0.1401 |
|  | rs501192 | G>A | 413 | 1 | 192 | 0 | 1.40 | 0.06-34.47 | 1 |
|  | rs530537 | A>G | 304 | 108 | 127 | 63 | 0.72 | 0.49-1.04 | 0.0811 |
|  | rs61751523 | T>C | 392 | 20 | 181 | 11 | 0.84 | 0.39-1.79 | 0.6932 |

HP, *Helicobacter pylori*; OR, odds ratio; CI, confidence intervals.

^*^ Fisher’s exact test two-tailed *P*-value.

**Table S7.** Effect of *Helicobacter pylori* infection on the expression of genes involved in the NOD-like receptors signalling pathway.

| **Gene symbol** | **Gene name** | ***Helicobacter pylori* GC026** | | | ***Helicobacter pylori* 26695** | | |
| --- | --- | --- | --- | --- | --- | --- | --- |
|  |  | **Median fold change** | **95% CI** | ***P-*value** | **Median fold change** | **95% CI** | ***P-*value** |
| *AIM2* | Absent in melanoma 2 | 0.07 | ( 0.00001, 0.15 ) | 0.0016 | 0.06 | ( 0.01, 0.11 ) | 0.0110 |
| *BCL2* | B-cell CLL/lymphoma 2 | 0.70 | ( 0.50, 0.90 ) | 0.0475 | 0.29 | ( 0.00001, 0.62 ) | 0.1477 |
| *BCL2L1* | BCL2-like 1 | 0.09 | ( 0.02, 0.16 ) | 0.0332 | 0.23 | ( 0.00001, 0.56 ) | 0.1923 |
| *BIRC2* | Baculoviral IAP repeat containing 2 | 1.27 | ( 1.08, 1.47 ) | 0.0381 | 0.71 | ( 0.00001, 2.27 ) | 0.3765 |
| *BIRC3* | Baculoviral IAP repeat containing 3 | 12.29 | ( 8.37, 16.20 ) | 0.0016 | 0.99 | ( 0.00001, 2.11 ) | 0.7263 |
| *CARD18* | Caspase recruitment domain family, member 18 | 1.02 | ( 0.79, 1.24 ) | 0.8651 | 1.59 | ( 0.00001, 4.56 ) | 0.4382 |
| *CARD6* | Caspase recruitment domain family, member 6 | 0.29 | ( 0.18, 0.41 ) | 0.0029 | 0.22 | ( 0.00001, 0.59 ) | 0.1449 |
| *CASP1* | Caspase 1, apoptosis-related cysteine peptidase | 1.04 | ( 0.85, 1.22 ) | 0.7008 | 0.32 | ( 0.12, 0.52 ) | 0.0182 |
| *CASP4* | Caspase 4, apoptosis-related cysteine peptidase | 0.54 | ( 0.38, 0.71 ) | 0.0093 | 0.21 | ( 0.11, 0.32 ) | 0.0220 |
| *CASP5* | Caspase 5, apoptosis-related cysteine peptidase | 3.09 | ( 2.63, 3.55 ) | 0.0004 | 1.00 | ( 1.00, 1.00 ) | 0.0000 |
| *CASP8* | Caspase 8, apoptosis-related cysteine peptidase | 0.28 | ( 0.20, 0.36 ) | 0.0054 | 0.06 | ( 0.00001, 0.17 ) | 0.0060 |
| *CCL2* | Chemokine (C-C motif) ligand 2 | 1.40 | ( 1.02, 1.79 ) | 0.0737 | 0.12 | ( 0.05, 0.19 ) | 0.0272 |
| *CCL5* | Chemokine (C-C motif) ligand 5 | 2.28 | ( 1.30, 3.26 ) | 0.0366 | 0.94 | ( 0.00001, 2.19 ) | 0.9940 |
| *CCL7* | Chemokine (C-C motif) ligand 7 | 0.59 | ( 0.45, 0.73 ) | 0.0124 | 0.11 | ( 0.00001, 0.24 ) | 0.0417 |
| *CD40LG* | CD40 ligand | 0.05 | ( 0.04, 0.06 ) | 0.0000 | 2.15 | ( 0.00001, 7.38 ) | 0.3977 |
| *CFLAR* | CASP8 and FADD-like apoptosis regulator | 1.74 | ( 0.77, 2.72 ) | 0.1168 | 0.36 | ( 0.01, 0.71 ) | 0.1734 |
| *CHUK* | Conserved helix-loop-helix ubiquitous kinase | 0.86 | ( 0.32, 1.41 ) | 0.4720 | 0.20 | ( 0.05, 0.35 ) | 0.0223 |
| *CIITA* | Class II, major histocompatibility complex, transactivator | 1.03 | ( 0.42, 1.64 ) | 0.8773 | 1.02 | ( 0.00001, 2.22 ) | 0.6540 |
| *CTSB* | Cathepsin B | 0.44 | ( 0.42, 0.46 ) | 0.0000 | 0.15 | ( 0.08, 0.22 ) | 0.0013 |
| *CXCL1* | Chemokine (C-X-C motif) ligand 1 | 22.45 | ( 8.83, 36.08 ) | 0.0006 | 2.95 | ( 0.75, 5.14 ) | 0.0188 |
| *CXCL2* | Chemokine (C-X-C motif) ligand 2 | 60.08 | ( 27.53, 92.64 ) | 0.0002 | 20.73 | ( 0.00001, 42.73 ) | 0.0575 |
| *FADD* | Fas (TNFRSF6)-associated via death domain | 0.10 | ( 0.00001, 0.20 ) | 0.0255 | 0.04 | ( 0.00001, 0.15 ) | 0.1890 |
| *HSP90AA1* | Heat shock protein 90kDa alpha (cytosolic), class A member 1 | 0.63 | ( 0.56, 0.71 ) | 0.0031 | 0.16 | ( 0.10, 0.22 ) | 0.0003 |
| *HSP90AB1* | Heat shock protein 90kDa alpha (cytosolic), class B member 1 | 0.96 | ( 0.55, 1.38 ) | 0.7407 | 0.11 | ( 0.01, 0.22 ) | 0.0204 |
| *HSP90B1* | Heat shock protein 90kDa beta (Grp94), member 1 | 0.42 | ( 0.26, 0.58 ) | 0.0040 | 0.19 | ( 0.05, 0.33 ) | 0.0894 |
| *IFNB1* | Interferon, beta 1, fibroblast | 32.50 | ( 15.41, 49.58 ) | 0.0001 | 3.49 | ( 0.57, 6.41 ) | 0.0642 |
| *IFNG* | Interferon, gamma | 1.02 | ( 0.79, 1.24 ) | 0.8651 | 0.27 | ( 0.00001, 0.91 ) | 0.3653 |
| *IKBKB* | Inhibitor of kappa light polypeptide gene enhancer in B-cells, kinase beta | 0.65 | ( 0.42, 0.87 ) | 0.0970 | 0.10 | ( 0.03, 0.17 ) | 0.0364 |
| *IKBKG* | Inhibitor of kappa light polypeptide gene enhancer in B-cells, kinase gamma | 0.21 | ( 0.00001, 0.48 ) | 0.1301 | 0.17 | ( 0.01, 0.34 ) | 0.0869 |
| *IL12A* | Interleukin 12A | 3.59 | ( 2.49, 4.69 ) | 0.0003 | 0.60 | ( 0.00001, 1.32 ) | 0.4203 |
| *IL12B* | Interleukin 12B | 434.20 | ( 0.00001, 1093.32 ) | 0.0399 | 5.36 | ( 0.00001, 17.63 ) | 0.2003 |
| *IL18* | Interleukin 18 | 0.43 | ( 0.29, 0.57 ) | 0.0072 | 0.12 | ( 0.02, 0.22 ) | 0.0694 |
| *IL1B* | Interleukin 1, beta | 3.70 | ( 1.56, 5.83 ) | 0.0086 | 63.26 | ( 0.00001, 478.44 ) | 0.0446 |
| *IL33* | Interleukin 33 | 4.78 | ( 3.81, 5.74 ) | 0.0000 | 15.82 | ( 0.00001, 43.71 ) | 0.0522 |
| *IL6* | Interleukin 6 | 262.39 | ( 32.53, 492.24 ) | 0.0008 | 94.57 | ( 0.00001, 234.99 ) | 0.0292 |
| *IRAK1* | Interleukin-1 receptor-associated kinase 1 | 0.08 | ( 0.00001, 0.17 ) | 0.0591 | 0.04 | ( 0.00001, 0.14 ) | 0.1675 |
| *IRF1* | Interferon regulatory factor 1 | 0.32 | ( 0.00001, 0.68 ) | 0.1060 | 0.50 | ( 0.00001, 1.10 ) | 0.4528 |
| *IRF2* | Interferon regulatory factor 2 | 0.21 | ( 0.13, 0.28 ) | 0.0108 | 0.09 | ( 0.01, 0.17 ) | 0.0796 |
| *MAP3K7* | Mitogen-activated protein kinase kinase kinase 7 | 0.54 | ( 0.44, 0.64 ) | 0.0071 | 0.15 | ( 0.07, 0.23 ) | 0.0053 |
| *TAB1* | TGF-beta activated kinase 1/MAP3K7 binding protein 1 | 0.09 | ( 0.05, 0.14 ) | 0.0021 | 0.09 | ( 0.00001, 0.19 ) | 0.1703 |
| *TAB2* | TGF-beta activated kinase 1/MAP3K7 binding protein 2 | 0.39 | ( 0.29, 0.50 ) | 0.0093 | 0.12 | ( 0.00001, 0.27 ) | 0.2276 |
| *MAPK1* | Mitogen-activated protein kinase 1 | 0.18 | ( 0.09, 0.26 ) | 0.0047 | 0.12 | ( 0.03, 0.21 ) | 0.0546 |
| *MAPK11* | Mitogen-activated protein kinase 11 | 0.91 | ( 0.39, 1.43 ) | 0.7202 | 0.08 | ( 0.00001, 0.17 ) | 0.0459 |
| *MAPK12* | Mitogen-activated protein kinase 12 | 0.16 | ( 0.09, 0.23 ) | 0.0050 | 0.12 | ( 0.00001, 0.52 ) | 0.2075 |
| *MAPK13* | Mitogen-activated protein kinase 13 | 0.34 | ( 0.18, 0.51 ) | 0.0560 | 0.11 | ( 0.01, 0.22 ) | 0.0191 |
| *MAPK3* | Mitogen-activated protein kinase 3 | 0.22 | ( 0.05, 0.39 ) | 0.0560 | 0.05 | ( 0.00001, 0.14 ) | 0.0325 |
| *MAPK8* | Mitogen-activated protein kinase 8 | 0.40 | ( 0.10, 0.70 ) | 0.0318 | 0.00 | ( 0.00001, 0.03 ) | 0.3739 |
| *MAPK9* | Mitogen-activated protein kinase 9 | 0.30 | ( 0.25, 0.35 ) | 0.0002 | 0.14 | ( 0.07, 0.21 ) | 0.0141 |
| *MEFV* | Mediterranean fever | 0.25 | ( 0.15, 0.36 ) | 0.0090 | 0.17 | ( 0.00001, 0.55 ) | 0.1632 |
| *MYD88* | Myeloid differentiation primary response gene (88) | 0.11 | ( 0.05, 0.17 ) | 0.0096 | 0.13 | ( 0.00001, 0.29 ) | 0.1700 |
| *NAIP* | NLR family, apoptosis inhibitory protein | 0.43 | ( 0.15, 0.70 ) | 0.0833 | 0.06 | ( 0.00001, 0.18 ) | 0.0926 |
| *NFKB1* | Nuclear factor of kappa light polypeptide gene enhancer in B-cells 1 | 2.50 | ( 1.90, 3.10 ) | 0.0068 | 0.58 | ( 0.00001, 1.25 ) | 0.4679 |
| *NFKBIA* | Nuclear factor of kappa light polypeptide gene enhancer in B-cells inhibitor, alpha | 6.60 | ( 4.38, 8.82 ) | 0.0001 | 1.46 | ( 0.43, 2.49 ) | 0.3920 |
| *NFKBIB* | Nuclear factor of kappa light polypeptide gene enhancer in B-cells inhibitor, beta | 0.38 | ( 0.14, 0.61 ) | 0.0979 | 0.05 | ( 0.00001, 0.17 ) | 0.0837 |
| *NLRC4* | NLR family, CARD domain containing 4 | 0.12 | ( 0.07, 0.17 ) | 0.0003 | 0.06 | ( 0.01, 0.10 ) | 0.0357 |
| *NLRC5* | NLR family, CARD domain containing 5 | 0.14 | ( 0.10, 0.18 ) | 0.0003 | 0.04 | ( 0.02, 0.06 ) | 0.0131 |
| *NLRP1* | NLR family, pyrin domain containing 1 | 0.40 | ( 0.00001, 1.23 ) | 0.9838 | 1.31 | ( 0.00001, 3.26 ) | 0.5062 |
| *NLRP12* | NLR family, pyrin domain containing 12 | 0.03 | ( 0.01, 0.04 ) | 0.0163 | 0.11 | ( 0.00, 0.21 ) | 0.1137 |
| *NLRP3* | NLR family, pyrin domain containing 3 | 0.67 | ( 0.32, 1.03 ) | 0.2053 | 0.21 | ( 0.00001, 0.50 ) | 0.1306 |
| *NLRP4* | NLR family, pyrin domain containing 4 | 1.02 | ( 0.79, 1.24 ) | 0.8651 | 1.97 | ( 0.00001, 6.41 ) | 0.4057 |
| *NLRP5* | NLR family, pyrin domain containing 5 | 1.02 | ( 0.79, 1.24 ) | 0.8651 | 1.62 | ( 0.00001, 4.68 ) | 0.4347 |
| *NLRP6* | NLR family, pyrin domain containing 6 | 1.02 | ( 0.79, 1.24 ) | 0.8651 | 0.94 | ( 0.00001, 3.07 ) | 0.5625 |
| *NLRP9* | NLR family, pyrin domain containing 9 | 0.77 | ( 0.30, 1.24 ) | 0.4548 | 0.12 | ( 0.00001, 0.26 ) | 0.0005 |
| *NLRX1* | NLR family member X1 | 0.02 | ( 0.00001, 0.05 ) | 0.0176 | 0.22 | ( 0.00001, 0.85 ) | 0.4216 |
| *NOD2* | Nucleotide-binding oligomerization domain containing 2 | 0.63 | ( 0.48, 0.78 ) | 0.0165 | 0.49 | ( 0.03, 0.95 ) | 0.2132 |
| *P2RX7* | Purinergic receptor P2X, ligand-gated ion channel, 7 | 6.99 | ( 5.17, 8.81 ) | 0.0017 | 3.10 | ( 0.00001, 7.12 ) | 0.2016 |
| *PANX1* | Pannexin 1 | 0.66 | ( 0.39, 0.93 ) | 0.1530 | 0.24 | ( 0.00001, 0.57 ) | 0.2751 |
| *PEA15* | Phosphoprotein enriched in astrocytes 15 | 0.49 | ( 0.00001, 1.07 ) | 0.2752 | 0.27 | ( 0.11, 0.42 ) | 0.0112 |
| *PSTPIP1* | Proline-serine-threonine phosphatase interacting protein 1 | 0.13 | ( 0.07, 0.19 ) | 0.0066 | 0.03 | ( 0.00001, 0.06 ) | 0.0541 |
| *PTGS2* | Prostaglandin-endoperoxide synthase 2 | 54.03 | ( 10.88, 97.17 ) | 0.0247 | 19.56 | ( 7.32, 31.81 ) | 0.0161 |
| *PYCARD* | PYD and CARD domain containing | 0.05 | ( 0.03, 0.07 ) | 0.0001 | 0.03 | ( 0.01, 0.05 ) | 0.0068 |
| *PYDC1* | PYD (pyrin domain) containing 1 | 1.00 | ( 0.77, 1.23 ) | 0.9658 | 0.80 | ( 0.26, 1.33 ) | 0.5153 |
| *RAGE* | Renal tumor antigen | 0.48 | ( 0.00001, 0.99 ) | 0.2113 | 0.04 | ( 0.00, 0.08 ) | 0.0450 |
| *RELA* | V-rel reticuloendotheliosis viral oncogene homolog A (avian) | 0.49 | ( 0.32, 0.67 ) | 0.0447 | 0.26 | ( 0.00001, 0.52 ) | 0.1310 |
| *RIPK2* | Receptor-interacting serine-threonine kinase 2 | 3.09 | ( 2.26, 3.92 ) | 0.0035 | 0.67 | ( 0.13, 1.21 ) | 0.4007 |
| *SUGT1* | SGT1, suppressor of G2 allele of SKP1 (S. cerevisiae) | 0.52 | ( 0.43, 0.60 ) | 0.0006 | 0.21 | ( 0.01, 0.42 ) | 0.0470 |
| *TIRAP* | Toll-interleukin 1 receptor (TIR) domain containing adaptor protein | 0.19 | ( 0.02, 0.36 ) | 0.0602 | 0.08 | ( 0.00001, 0.27 ) | 0.2154 |
| *TNF* | Tumor necrosis factor | 32.12 | ( 19.19, 45.05 ) | 0.0003 | 14.32 | ( 7.45, 21.19 ) | 0.0108 |
| *TNFSF11* | Tumor necrosis factor (ligand) superfamily, member 11 | 0.13 | ( 0.00001, 0.26 ) | 0.0092 | 0.48 | ( 0.00001, 1.69 ) | 0.3440 |
| *TNFSF14* | Tumor necrosis factor (ligand) superfamily, member 14 | 0.16 | ( 0.00001, 0.36 ) | 0.0925 | 0.07 | ( 0.00001, 0.24 ) | 0.2144 |
| *TNFSF4* | Tumor necrosis factor (ligand) superfamily, member 4 | 0.53 | ( 0.00001, 1.37 ) | 0.2855 | 0.25 | ( 0.00001, 0.83 ) | 0.4085 |
| *TRAF6* | TNF receptor-associated factor 6 | 0.64 | ( 0.02, 1.27 ) | 0.3091 | 0.18 | ( 0.04, 0.31 ) | 0.0678 |
| *TXNIP* | Thioredoxin interacting protein | 0.06 | ( 0.02, 0.10 ) | 0.0025 | 0.06 | ( 0.00001, 0.13 ) | 0.1814 |
| *XIAP* | X-linked inhibitor of apoptosis | 0.42 | ( 0.22, 0.62 ) | 0.0429 | 0.13 | ( 0.00001, 0.27 ) | 0.1044 |

CI, confidence intervals.
