# Supplementary material for: Correction: The NOD-Like Receptor Signalling Pathway in Helicobacter pylori Infection and Related Gastric Cancer: A Case-Control Study and Gene Expression Analyses
Source: PLoS One. 2015 Jan 30;10(1):e0117870. doi: 10.1371/journal.pone.0117870 (PMC4312023; doi:10.1371/journal.pone.0117870)
Supplement: S1 File — (PDF) [file pone.0117870.s001.pdf]

The NOD-Like Receptor Signalling Pathway in *Helicobacter pylori* Infection and Related Gastric Cancer: A Case-Control Study and Gene Expression Analyses

Natalia Castaño-Rodríguez[1], Nadeem O. Kaakoush[1], Khean-Lee Goh[2], Kwong Ming Fock[3], Hazel M. Mitchell[1]*

1 School of Biotechnology and Biomolecular Sciences, The University of New South Wales, Sydney, New South Wales, Australia, **2** Department of Medicine, Faculty of Medicine, University of Malaya, Kuala Lumpur, Malaysia, **3** Division of Gastroenterology, Department of Medicine, Changi General Hospital, Singapore, Singapore

Abstract

Background: Currently, it is well established that cancer arises in chronically inflamed tissue. A number of NOD-like receptors (NLRs) form inflammasomes, intracellular multiprotein complexes critical for generating mature pro-inflammatory cytokines (IL-1β and IL-18). As chronic inflammation of the gastric mucosa is a consequence of *Helicobacter pylori* infection, we investigated the role of genetic polymorphisms and expression of genes involved in the NLR signalling pathway in *H. pylori* infection and related gastric cancer (GC).

Materials and Methods: Fifty-one genetic polymorphisms were genotyped in 310 ethnic Chinese (87 non-cardia GC cases and 223 controls with functional dyspepsia). In addition, gene expression of 84 molecules involved in the NLR signalling pathway was assessed in THP-1 cells challenged with two *H. pylori* strains, GC026 (GC) and 26695 (gastritis).

Results: *CARD8*-rs11672725, *NLRP3*-rs10754558, *NLRP3*-rs4612666, *NLRP12*-rs199475867 and *NLRX1*-rs10790286 showed significant associations with GC. On multivariate analysis, *CARD8*-rs11672725 remained a risk factor (OR: 4.80, 95% CI: 1.39–16.58). Further, *NLRP12*-rs2866112 increased the risk of *H. pylori* infection (OR: 2.13, 95% CI: 1.22–3.71). Statistical analyses assessing the joint effect of *H. pylori* infection and the selected polymorphisms revealed strong associations with GC (*CARD8*, *NLRP3*, *CASP1* and *NLRP12* polymorphisms). In gene expression analyses, five genes encoding NLRs were significantly regulated in *H. pylori*-challenged cells (*NLRC4*, *NLRC5*, *NLRP9*, *NLRP12* and *NLRX1*). Interestingly, persistent up-regulation of *NFKB1* with simultaneous down-regulation of *NLRP12* and *NLRX1* was observed in *H. pylori* GC026-challenged cells. Further, NF-κB target genes encoding pro-inflammatory cytokines, chemokines and molecules involved in carcinogenesis were markedly up-regulated in *H. pylori* GC026-challenged cells.

Conclusions: Novel associations between polymorphisms in the NLR signalling pathway (*CARD8*, *NLRP3*, *NLRP12*, *NLRX1*, and *CASP1*) and GC were identified in Chinese individuals. Our genetic polymorphisms and gene expression results highlight the relevance of the NLR signalling pathway in gastric carcinogenesis and its close interaction with NF-κB.

Citation: Castaño-Rodríguez N, Kaakoush NO, Goh K-L, Fock KM, Mitchell HM (2014) The NOD-Like Receptor Signalling Pathway in *Helicobacter pylori* Infection and Related Gastric Cancer: A Case-Control Study and Gene Expression Analyses. PLoS ONE 9(6): e98899. doi:10.1371/journal.pone.0098899

Editor: David M. Ojcius, University of California Merced, United States of America

Received March 2, 2014; **Accepted** May 8, 2014; **Published** June 5, 2014

Copyright: © 2014 Castaño-Rodríguez et al. This is an open-access article distributed under the terms of the Creative Commons Attribution License, which permits unrestricted use, distribution, and reproduction in any medium, provided the original author and source are credited.

Funding: This work was supported in part by The Cancer Council of New South Wales, Australia (Grant no.66/04). N.O. Kaakoush is supported by an Early Career fellowship from the National Health and Medical Research Council, Australia. The funders had no role in study design, data collection and analysis, decision to publish, or preparation of the manuscript.

Competing Interests: The authors have declared that no competing interests exist.

* E-mail: h.mitchell@unsw.edu.au

Introduction

The overall incidence of gastric cancer (GC) ranges widely among countries. According to global cancer statistics, a total of 952,000 new GC cases and 723,000 GC-related deaths are estimated to have occurred in 2012, accounting for 6.8% of the total cancer cases and 8.8% of total cancer-related deaths [1]. Over 70% of new cases and deaths occur in developing countries, with the highest incidence rates being reported in East Asia, Eastern Europe, and Central and South America [2].

The bacterial pathogen *Helicobacter pylori* is an essential aetiological factor for GC (IARC, 1994), however, previous studies suggest that in addition to *H. pylori* infection and dietary factors, host genetics contribute to GC [3]. Genetic polymorphisms have emerged in recent years as determinants of disease susceptibility and severity, which is particularly true in gastrointestinal malignancy [4]. Therefore, polymorphisms within genes involved in innate and adaptive immunity might play an important role in the pathogenesis of *H. pylori* infection and development of *H. pylori*-related complications including GC.

Germ-line encoded receptors known as pattern-recognition receptors (PRRs) are part of the innate immune system and are pivotal for the detection of invariant microbial motifs known as pathogen-associated molecular patterns (PAMPs). PRRs have been divided into five distinct genetic and functional clades: nucleotide-binding oligomerization domain (NOD)-like receptors

(NLRs), Toll-like receptors, C-type lectin receptors, retinoic acid-inducible gene (RIG)-I-like receptors and absent in melanoma 2 (AIM2)-like receptors [5] [6].

The NLR family not only recognise PAMPs but also damage-associated molecular patterns (DAMPs) in the cytoplasm, which are endogenous ligands produced after tissue injury or cell death [7]. The NLRs characteristic structure includes a central nucleotide-binding and oligomerization (NACHT) domain that is present in all NLR family members, a C-terminal leucine-rich repeats (LRRs) and an N-terminal caspase recruitment (CARD) or pyrin (PYD) domain. Based on phylogenetic analysis of NACHT domains, it was determined that the NLR family comprises three subfamilies: 1) the NOD family which includes NOD1–2, NOD 3/NLRC3, NOD4/NLRC5, NOD5/NLRX1 and CIITA, 2) the NLRPs including NLRP1–14 (also known as NALPs), and 3) the IPAF subfamily which consists of IPAF (NLRC4) and NAIP [7]. The NLRP3 inflammasome, the most fully characterized inflammasome, consists of the NLRP3 scaffold protein, the apoptosis associated speck-like protein (ASC) and caspase-1. NLRP3 interacts and recruits the adaptor ASC via PYD-PYD interaction [8]. This interaction leads to the recruitment of caspase-1, an intracellular aspartate specific cysteine protease, which would subsequently lead to the maturation and release of proinflammatory cytokines such as IL-1β and IL-18.

In *H. pylori* infection, the first four physical-chemical barriers are the mucus layer, gastric epithelial cells, the TLRs and the NLRs. A limited number of studies have assessed the interaction between the NLR signalling pathway and *H. pylori*. For example, an initial study by Basak et al. [9] showed that not only *H. pylori* lipopolysaccharide (LPS) activates caspase-1 but that this caspase-1 activation is involved in LPS-induced IL-1β maturation. Later, Hitzler et al. [10] showed that *H. pylori* infection activates caspase-1, leading to IL-1β/IL-18 processing and secretion, both in cultured dendritic cells (DCs) and *in vivo*. Consistently, two recent studies, using human gastric cell lines, confirmed increased expression of caspase-1, IL-1β and IL-18 in *H. pylori*-infected cells [11,12]. Further, a recent study by Kim et al. [13] has shown that secretion of IL-1β by DCs infected with *H. pylori* requires TLR2, NOD2 and the NLRP3 inflammasome.

Therefore, previous studies clearly show that, in response to *H. pylori*, inflammasome-dependent caspase-1 activation is critical for generating mature pro-inflammatory cytokines that are crucial for Th1 responses associated with gastric immunopathology. Given that little is known about the role of inflammasomes and other molecules involved in the NLR signalling pathway in response to *H. pylori* infection, and that functionally relevant polymorphisms in genes of this arm of the immune system have the potential to affect the magnitude and direction of the host response against the infection, we investigated the role of the NLR signalling pathway, including inflammasome-related molecules, in *H. pylori*-related GC development by assessing 51 genetic polymorphisms in Chinese individuals, a known high risk population for GC, and addressing the gene expression of 84 molecules involved in NLR signalling pathways in a monocytic cell line upon exposure to *H. pylori*.

Materials and Methods

Genotyping of Polymorphisms Involved in the NOD-like Receptor Signalling Pathway

Ethics statement. This study was approved by the Human Ethics Committee (HREC) of the University of New South Wales (UNSW) (HREC 08115 and HREC 02144). Written informed consent was obtained from each individual recruited to the study.

Study subjects. Subjects were ethnic Chinese individuals who underwent upper gastrointestinal endoscopy at the Changi General Hospital (Singapore) and the University Hospital of Malaysia (Kuala Lumpur), between January 2004 and April 2007. Patients known to be infected with the human immunodeficiency virus or who had been prescribed non-steroidal anti-inflammatory drugs, anti-microbial agents or acid suppressants in the two-month period prior to recruitment were excluded.

Eighty-seven patients newly diagnosed with a primary non-cardia GC (International Classification of Diseases, 9th revision, code 151) based on histological confirmation were invited to participate in the study. The control group comprised 223 individuals diagnosed with functional dyspepsia (FD), which was defined as persistent or recurrent symptoms (pain or discomfort centred in the upper abdomen) in the absence of organic disease (including at upper endoscopy), in accordance with Rome II classification system [14].

***Helicobacter pylori* detection.** *H. pylori* IgG antibodies in these Chinese individuals were determined using an in-house enzyme-linked immunosorbent assay [15] and immunoblot (MPD Helico Blot 2.1, MP Biomedicals, Australia).

Polymorphisms selection. Electronic databases (PUBMED, Scopus, Science Direct, Ovid, Biosis Previews, Scirus databases, CINAHL, IMBIOMED, Scielo and LILACS) were searched up to February 2013 for polymorphisms involved in the NLR signalling pathway that were associated with cancer, infectious disease or were functionally relevant. Fifty one polymorphisms in 6 genes, which were reported to have a minor allele frequency >1% in the National Center for Biotechnology Information (NCBI) dbSNP, were selected for analysis (Table S1 in Tables S1).

Genotyping techniques. For genotyping of the 51 selected polymorphisms in the NLR signalling pathway of each individual included in the study, genomic DNA was extracted from peripheral whole blood samples using the QIAamp Blood DNA Mini Kit as described by the manufacturer (Qiagen; Chadstone, Australia). DNA was rehydrated in sterile water and normalised to 10 ng/µl for customized SNP genotyping through the application of matrix assisted laser desorption ionization time-of-flight (MALDI-TOF) mass spectrometry, the Sequenom MassARRAY iPLEX?assay (San Diego, CA, USA) [16,17] at the Australian Genome Research Facility Ltd, St Lucia, University of Queensland, Australia.

One polymorphism that could not be included in the Sequenom MassARRAY iPLEX assay, known as *NLRP3*–42 bp-VNTR, was genotyped through standard PCR. Primers were designed with Oligo Primer Analysis Software version 6.71 (Molecular Biology Insights, Inc; Colorado Springs, USA). Experiments were performed using the 2720 Thermal Cycler (Applied Biosystems; Foster City, USA). PCR products were subjected to 1.5% agarose gel electrophoresis and visualised under UV transillumination using the Gel Doc 2000 System (Bio-Rad; Hercules, USA). The primer sequences and thermal cycling conditions for genotyping of this polymorphism are described in Table S2 in in Tables S1.

Four polymorphisms (*CARD8*-rs2043211, *CARD8*-rs6509368, *CARD8*-rs12984929 and *NLRP3*-rs10754558) genotyped by MALDI-TOF mass spectrometry were randomly selected for confirmation of the results using real-time PCR. The primer sequences and thermal cycling conditions are outlined in Table S2 in Tables S1. As a validation of the methodology implemented for the genotyping of *NLRP3*–42 bp-VNTR, sequencing analyses were performed in 10% of randomly selected study samples, at the Ramaciotti Centre, UNSW, Australia.

Statistical analysis. The unpaired Student's *t*-test was used to analyse the clinical characteristics. The direct count method was used to estimate the genotype and allele frequencies. Deviation from Hardy-Weinberg equilibrium (HWE) was tested using the chi-square goodness-of-fit test (χ^2). Determination of linkage disequilibrium (LD) was based on a likelihood ratio test in which the significance of the observed likelihood ratio is found by computing the null distribution of this ratio under the hypothesis of linkage equilibrium, using a permutation procedure [18]. Haplotype inference was performed by means of the Expectation-Maximization (EM) algorithm for multi-locus genotypic data [19]. The odds ratios (OR) and 95% confidence intervals (CI) were calculated by means of the Fisher's exact probability test (two-tailed *p*-values). In order to correct for confounding factors, a binary logistic regression (LR) adjusted by *H. pylori* status and gender was performed. *P*-values$<$0.05 were considered as statistically significant. Quality filters for exclusion of polymorphisms from the statistical analysis included call rates below 95% and deviation from HWE. The data was analysed by means of the programs Arlequin version 3.1 [20], GraphPad Prism version 5.02 (GraphPad Software Inc; San Diego, USA) and SPSS version 19.0.0 (SPSS Inc; Chicago, USA).

Gene Expression of Molecules Involved in the NOD-like Receptor Signalling Pathway

Mammalian cell culture. The human monocytic leukaemia cell line THP-1 (code: TIB-202) (American Type Culture Collection; Manassas, USA), was cultured in RPMI 1640 medium supplemented with 10% heat-inactivated fetal bovine serum (FBS) (Invitrogen; Mulgrave, Australia), 1 mM sodium pyruvate (Invitrogen), 2500 mg/L sodium bicarbonate (Invitrogen) and 100 μg/mL penicillin-streptomycin solution (Invitrogen). Cells were maintained in 25-cm^2 tissue culture flasks (Greiner-Bio-On; Frickenhausen, Germany) at 37°C with 5% CO_2.

Bacterial culture. The *H pylori* strain GC026 (*cagA*+, *cagE*+, *cagL*+, *cagT*+, *vacA* s1 m1+, *babA*+, *oipA*+, *dupA*+ and *sabA*+) was isolated from a GC patient at the University Hospital of Malaysia, Kuala Lumpur [21,22]. The *H. pylori* strain 26695 (*cagA*+ and *vacA* s1m1+) was isolated from a patient with gastritis [23] (ATCC code 700392). Both *H. pylori* strains were grown for two days on horse blood agar plates (Blood Agar Base No.2 supplemented with 6% sterile defibrinated horse blood (Oxoid, Heidelberg West, Vic., Australia) at 37°C in an anaerobic jar containing a gas generating kit (Oxoid) to provide a microaerobic atmosphere of 6% O_2, 10% CO_2 and 84% N_2.

Infection assay. THP-1 cells were seeded in a 6-well culture plate at a concentration of 5×10^5 cells/ml and subsequently differentiated into macrophages with phorbol myrastate acetate (Sigma-Aldrich) for 72 hours [24]. Prior to bacterial infection, mammalian cells were incubated in antibiotic-free medium. The *H. pylori* strains GC026 and 26695 were then added to the medium at a multiplicity of infection of 1, and incubated for 6 hours at 37°C and 5% CO_2. The bacterial concentration added was determined based on a standard curve and optical density (OD) readings at 595 nm using a Bio-Rad microplate reader model 550 (Bio-Rad). The actual concentration added was confirmed by counting colony forming units (CFU) grown on horse blood agar plates after serial dilution of the bacterial suspension.

RNA extraction, cDNA preparation and PCR arrays. After infection, non-challenged and *H. pylori*-challenged THP-1 cells were used for the isolation of mRNA using the Qiagen RNeasy Mini Kit as described by the manufacturer (Qiagen). For the analysis of the gene expression of 84 molecules involved in the NLR signalling pathway, cDNA was synthesised from 0.8 μg total RNA using the RT^2 First Strand cDNA Kit (Qiagen) and further analysed using the Human Inflammasome RT^2 Profiler PCR array (PAHS-097R) (Qiagen) as recommended by manufacturer. Gene expression profiles were obtained from three independent experiments of *H. pylori* GC026-infected, *H. pylori* 26695-infected and corresponding non-infected (control) samples. The experiments were performed employing the Rotor-Gene Q cycler (Corbett Life Sciences; Doncaster, Australia).

For gene expression data analysis, the ΔΔ Ct-based method of relative quantification was implemented using the Web-based RT^2 Profiler PCR Array Data Analysis version 3.5 (http://pcrdataanalysis.sabiosciences.com/pcr/arrayanalysis.php). At least two-fold changes ($\geq$2, $\leq$0.5) and *P*-values$<$0.05 were considered as statistically significant.

SDS-PAGE and Western Blotting

THP-1 cells were seeded, differentiated and infected at a MOI of 1, as previously described. Proteins were extracted using RIPA buffer, separated on 12% sodium dodecyl sulfate polyacrylamide gel electrophoresis gels, and transferred to methanol-treated polyvinylidine difluoride membranes using the Trans-blot cell transfer system (Bio-Rad). Membranes were immunolabeled with rabbit anti-human polyclonal antibodies against NLRX1 (1:200) or mouse anti-human monoclonal antibodies against β-actin (1:1000) (Santa Cruz). Goat anti-rabbit and anti-mouse IgG antibodies coupled to HRP (1:2000; Bio-Rad) were used as secondary antibodies, respectively. Membranes were probed in accordance with the Pierce ECL Western Blotting Substrate protocol (Thermo Scientific; Scoresby, Australia).

Results

Clinical Characteristics

The clinical characteristics of the study subjects including gender, *H. pylori* infection status and median age are shown in Table S3 in Tables S1. Male gender was found to be more predominant in GC patients than in FD controls, showing a positive association with the development of GC in this ethnic Chinese population (OR: 2.15, 95% CI: 1.29–3.58, *P*-value: 0.0036). *H. pylori* infection was found to be present in 68.7% of individuals in this study (73/87 GC patients and 140/223 FD controls). Comparison of the prevalence of *H. pylori* infection in GC patients and FD controls showed that *H. pylori* infection was a risk factor for the development of GC (OR: 2.38, 95% CI: 1.41–3.99, *P*-value:$<$0.0001). Even though subjects in this study were matched according to 5-years age groups, the median age of GC patients (65.3 yrs) was significantly higher than that of FD controls (54.3 yrs) (*P*-value:$<$0.0001).

Polymorphisms in Genes Involved in the NOD-like Receptor Signalling Pathway are Associated with Gastric Cancer in Ethnic Chinese Individuals

Fifty-one polymorphisms in genes involved in the NLR signalling pathway were genotyped in 310 ethnic Chinese individuals (87 GC cases and 223 FD controls). The call rate of all the selected polymorphisms was 97–100% in this study. All polymorphisms in the NLR signalling pathway were found to be in HWE in the control group showing non-significant χ^2 values except for *CARD8*-rs12984929 (χ^2: 35.98), *CARD8*-rs4802445 (χ^2: 5.87) and *CARD8*-rs6509368 (χ^2: 5.43). Twenty one polymorphisms in *NLRP3* (n = 5), *NLRP12* (n = 3), *NLRX1* (n = 3), *ASC* (n = 4) and *CASP1* (n = 6) were found to be non-polymorphic in this ethnic Chinese population (Table S1 in in Tables S1).

Allele and genotype frequencies as well as ORs and 95% CI for the remaining 27 polymorphisms are shown in Table 1 and Table S4 in Tables S1. The *CARD8*-rs11672725 TT genotype showed a strong association with GC in the bivariate statistical analysis (OR: 4.80, 95% CI: 1.39–16.58). In addition to *CARD8*-rs11672725, another four polymorphisms showed borderline results in the bivariate analysis (*NLRP3*-rs10754558, *NLRP3*-rs4612666, *NLRP12*-rs199475867 and *NLRX1*-rs10790286) (Table 1). Further multivariate statistical analyses, adjusting for *H. pylori* infection and male gender, showed that the *CARD8*-rs11672725 TT genotype remained a risk factor for GC in this ethnic Chinese population (Table 2).

Haplotype-based analyses are a powerful approach to dissect the architecture of complex diseases. In the current study, the EM algorithm identified 25 haplotypes with a frequency >0.01 in GC cases (Table S5 in Tables S1). Using the major haplotype in the control group as the reference haplotype, two *CARD8-NLRP12* (Hap1 and Hap8), one *NLRP3* (Hap3) and three *NLRX1-CASP1* (Hap21, 23 and 24) haplotypes were found to increase the risk of GC in this ethnic Chinese population. Interestingly, Hap1 harbours the *CARD8*-rs11672725 T allele, and Hap21 and Hap23 harbour the *NLRX1*-rs10790286 C allele, which shows consistency with the results obtained from the allele and genotype analyses.

Polymorphisms in Genes Involved in the NOD-like Receptor Signalling Pathway are Associated with *Helicobacter Pylori* Infection in Ethnic Chinese Individuals

Given that *H. pylori* is known to be a major risk factor associated with GC, further analyses were performed to assess the association between genetic polymorphisms involved in the NLR signalling pathway and *H. pylori* infection. Interestingly, our ethnic Chinese case-control study showed that *NLRX1*-rs10790286, *NLRP12*-rs2866112, *NLRP12*-rs4419163 and *ASC*-rs8056505 were associated with a significantly increased risk of *H. pylori* infection in these individuals (Table S6 in Tables S1). Multivariate statistical analysis confirmed that *NLRP12*-rs2866112 was associated with an increased risk of *H. pylori* in Chinese individuals (OR: 2.13, 95% CI: 1.22–3.71).

Joint Effect of *Helicobacter Pylori* Infection and Genetic Polymorphisms Markedly Increases the Risk of Gastric Cancer in Ethnic Chinese Individuals

Further analyses were performed to assess not only the presence or absence of the selected polymorphisms but also *H. pylori* status in relation to the risk of GC, in an attempt to determine the existence of biological interaction in the form of synergism or antagonism. Strikingly, individuals harbouring ten of the selected polymorphisms (*CARD8*-rs10405717, *NLRP3*-rs12079994, *NLRP3*-rs3806265, *NLRP3*-rs4612666, *NLRP12*-rs2866112, *NLRP12*-rs4419163, *NLRX1*-rs10790286, *CASP1*-rs2282659, *CASP1*-rs530537 and *CASP1*-rs61751523) and infected with *H. pylori* were observed to be at most risk of GC, the majority of these ORs being in the range 4.0–5.0 (Table 3). In contrast, in the absence of *H. pylori* infection, *CARD8*-rs2043211 significantly decreased the risk of GC (OR: 0.19, 95% CI: 0.06–0.63).

Helicobacter Pylori Influences the Expression of Several Molecules Involved in the NOD-like Receptor Signalling Pathway

To further characterise the effect of *H. pylori* on molecules involved in the NLR signalling pathway, we assessed the expression of 84 genes encoding NLRs, other inflammasome components, negative regulators, pro-inflammatory cytokines, pro-inflammatory caspases and molecules involved in downstream signalling, in THP-1-derived macrophages following exposure to two different *H. pylori* strains (GC026 and 26695). In *H. pylori* GC026-challenged THP1 cells, 49 genes were differentially expressed when compared to the control group (Table S7 in Tables S1and Figure S1 in Figures S1). Of these, statistically significant up-regulation and down-regulation of at least two-fold was found in 17 and 28 genes, respectively (Tables 4 and 5). Thirty five genes were differentially expressed between the control group and the group exposed to *H. pylori* 26695 (Table S7 in Tables S1 and Figure S2 in Figures S1). Of these, statistically significant up-regulation and down-regulation of at least two-fold was found in 5 and 23 genes, respectively (Tables 4 and 5).

Helicobacter Pylori Strains Associated with Gastric Cancer and Gastritis Lead to Different Expression Levels of Cytokines and Chemokines

Given that inflammation is a hallmark of gastric carcinogenesis, we further analysed the expression of pro-inflammatory cytokines and chemokines upon exposure to two *H. pylori* strains. The expression of nine genes encoding pro-inflammatory cytokines (*IFNB1*, *IL1B*, *IL12B*, *IL6*, *IL33* and *TNF*) and chemokines (*CXCL1*, *CXCL2*, *CCL5*) were significantly up-regulated in THP-1 cells challenged with both *H. pylori* GC026 and 26695 strains (Table 4). Further, an intense immune response (*CXCL1*, *CXCL2*, *CCL5*, *IL6*, *IL12B*, *TNF* and *IFNB1*) was initiated against *H. pylori* GC026 (Figure 1A). Interestingly, a number of genes encoding chemokines (*CCL2* and *CCL7*) and cytokines (*IL18*, *TNFSF11* and *CD40LG*) were down-regulated in *H. pylori*-challenged THP-1 cells (Table 5).

Helicobacter Pylori Infection Results in Up-regulation of *NFKB1* with Simultaneous Marked down-Regulation of *NLRP12* and *NLRX1*

Given that NF-κB is negatively regulated by NLRP12 and NLRX1, we further compared the expression of *NFKB1* and *RELA* between *H. pylori* GC026- and 26695-challenged cells. Remarkably, although *RELA* showed decreased levels in THP-1 cells exposed to both *H. pylori* strains (fold regulation: 0.49, *p*-value: 0.044673 and fold regulation: 0.26, *p*-value: 0.131017 for *H. pylori* GC026 and 26695, respectively), statistically significant up-regulation of *NFKB1* was only observed in *H. pylori* GC026-challenged cells (fold regulation: 2.50, *p*-value: 0.006825) (Figure 1C).

Helicobacter Pylori Increases the Expression of *PTGS2* and *BIRC3*

Expression of other molecules involved not only in the NLR signalling pathway but also in carcinogenesis was also analysed by comparing THP-1 cells responses to both *H. pylori* strains. Interestingly, *PTGS2* was significantly up-regulated in THP-1 cells upon exposure to both *H. pylori* strains, *H. pylori* GC026-challenged cells (fold regulation: 54.03, *p*-value: 0.0247) showing significantly higher expression levels when compared to *H. pylori* 26695-challenged cells (fold regulation: 19.56, *p*-value: 0.016089) (Figure 1d). Further, a second gene involved in carcinogenesis, *BIRC3*, was exclusively up-regulated in *H. pylori* GC026-challenged cells (fold regulation: 12.28, *p*-value: 0.001638) (Figure 1D).

Table 1. Association between polymorphisms involved in the NOD-like receptors signalling pathway and risk of gastric cancer in ethnic Chinese individuals (bivariate statistical analysis).

Gene	Polymorphism	Nucleotide change	Allele Analysis							Genotype Analysis											
			GC cases		FD controls		OR	95% CI	*P*-value*	GC cases			FD controls			XX vs YY			XX vs XY		
			X	Y	X	Y				XX	XY	YY	XX	XY	YY	OR	95% CI	*P*-value*	OR	95% CI	*P*-value*
CARD8	rs10405717	C>T	118	54	294	130	1.04	0.70–1.52	0.9221	40	38	8	98	98	16	1.23	0.49–3.09	0.6374	0.95	0.56–1.61	0.8939
	rs11670259	C>T	138	34	340	84	1.00	0.64–1.56	1	55	28	3	134	72	6	1.22	0.29–5.05	0.723	0.95	0.55–1.62	0.8918
	rs11672725	C>T	130	42	332	84	1.28	0.84–1.95	0.2701	53	24	9	129	74	5	**4.38**	**1.40–13.69**	**0.0134**	0.79	0.45–1.38	0.4833
	rs16981829	T>C	79	93	208	210	1.17	0.82–1.67	0.4156	19	41	26	57	94	58	1.35	0.68–2.70	0.482	1.31	0.69–2.47	0.4313
	rs1966625	G>A	142	30	336	88	0.81	0.51–1.28	0.4272	58	26	2	130	76	6	0.75	0.15–3.82	1	0.77	0.45–1.32	0.3471
	rs2043211	A>T	79	93	210	214	1.16	0.81–1.65	0.4695	21	37	28	54	102	56	1.29	0.65–2.53	0.4956	0.93	0.50–1.75	0.8725
	rs4802449	G>A	133	39	326	98	0.98	0.64–1.49	1	51	31	4	122	82	8	1.20	0.34–4.15	0.7522	0.90	0.53–1.53	0.7894
NLRP3	rs10754558	C>G	112	60	244	178	0.73	**0.51–1.06**	**0.1165**	38	36	12	74	96	41	0.57	0.27–1.21	0.1518	0.73	0.42–1.26	0.2674
	rs10925026	A>C	103	69	250	176	0.95	0.66–1.37	0.8543	31	41	14	72	106	35	0.93	0.44–1.97	1	0.90	0.52–1.56	0.7769
	rs12079994	G>A	149	23	373	53	1.09	0.64–1.84	0.7866	63	23	0	165	43	5	0.24	0.01–4.35	0.3273	1.40	0.78–2.51	0.2831
	rs1539019	G>T	103	69	247	177	0.93	0.65–1.34	0.783	30	43	13	70	107	35	0.87	0.40–1.87	0.847	0.94	0.54–1.63	0.8873
	rs3806265	T>C	81	91	223	203	1.23	0.87–1.76	0.2783	21	39	26	61	101	51	1.48	0.75–2.94	0.2987	1.12	0.60–2.08	0.7562
	rs4612666	C>T	79	91	227	199	**1.31**	**0.92–1.88**	**0.1467**	21	37	27	62	103	48	1.66	0.84–3.29	0.1675	1.06	0.57–1.97	0.876
	rs4925650	G>A	100	72	225	191	0.85	0.59–1.22	0.4121	33	34	19	67	91	50	0.77	0.39–1.51	0.5001	0.76	0.43–1.35	0.3802
NLRP12	rs104895565	A>del	171	1	433	3	0.84	0.09–8.18	1	85	1	0	215	3	0	2.52	0.05–128.20	1	0.84	0.09–8.22	1
	rs199475867	T>G	172	0	427	9	**0.13**	**0.01–2.26**	**0.0671**	86	0	0	209	9	0	2.42	0.05–123.20	1	**0.13**	**0.01–2.22**	**0.0651**
	rs2866112	C>G	132	40	333	101	1.00	0.66–1.52	1	51	30	5	128	77	12	1.05	0.35–3.12	1	0.98	0.57–1.67	1
	rs4419163	T>A	125	45	309	113	0.98	0.66–1.47	1	46	33	6	115	79	17	0.88	0.33–2.38	1	1.04	0.61–1.78	0.8927
	rs4539722	A>G	90	80	218	214	0.91	0.63–1.30	0.5884	24	42	19	58	102	56	0.82	0.41–1.66	0.5964	1.00	0.55–1.81	1
NLRX1	rs10790286	T>C	107	65	297	137	**1.32**	**0.91–1.90**	**0.1523**	33	41	12	107	83	27	1.44	0.66–3.16	0.4051	**1.60**	**0.93–2.75**	**0.1**
ASC	rs151056688	C>T	162	0	423	1	0.87	0.04–21.45	1	81	0	0	211	1	0	2.50	0.05–127.30	1	0.83	0.03–20.70	1
	rs8056505	T>C	120	52	320	116	1.20	0.81–1.76	0.3669	42	36	8	120	80	18	1.27	0.51–3.14	0.6351	1.29	0.76–2.18	0.4168
CASP1	rs2282659	A>G	123	49	316	116	1.09	0.73–1.61	0.6867	43	37	6	117	82	17	0.96	0.36–2.60	1	1.23	0.73–2.07	0.5037
	rs501192	G>A	172	0	435	1	0.84	0.03–20.77	1	86	0	0	217	1	0	2.51	0.05–127.90	1	0.84	0.03–20.79	1
	rs530537	A>G	123	49	310	122	1.01	0.68–1.50	1	43	37	6	117	76	23	0.71	0.27–1.86	0.6459	1.33	0.78–2.24	0.3449
	rs61751523	T>C	160	10	415	21	1.24	0.57–2.68	0.6815	76	8	1	197	21	0	7.75	0.31–192.40	0.281	0.99	0.42–2.33	1

GC, gastric cancer; FD, functional dyspepsia; X, wild allele; Y, mutant allele; XX, wild homozygote; XY, heterozygote; YY, mutant homozygote; OR, odds ratio; CI, confidence intervals.
*Fisher's exact test two-tailed *P*-value.
doi:10.1371/journal.pone.0098899.t001

Table 2. Association between identified risk factors and gastric cancer in ethnic Chinese individuals (binary logistic regression).

Variable	Adjusted OR	95% CI	*P*-value*
H. pylori infection	3.22	1.68–6.19	0.0001
Male gender	1.99	1.16–3.41	0.013
CARD8 rs11672725 TT	4.8	1.39–16.58	0.013

OR, odds ratio; CI, confidence intervals.
**P*-values were obtained from a binary logistic regression (backward Wald) adjusted by *Helicobacter pylori* status and gender.
doi:10.1371/journal.pone.0098899.t002

Discussion

GC is now considered a multifactorial process, in which bacterial, environmental and host genetics factors are involved at different stages in cancer pathophysiology. Currently, it is well established that cancer arises in chronically inflamed tissue, and this is particularly notable in the gastrointestinal tract [4]. As chronic inflammation of the gastric mucosa is a consequence of *H. pylori* infection and this bacterium is initially targeted by PRRs, we investigated the role of molecules involved in the NLR signalling pathway in *H. pylori* infection and related GC.

To our knowledge, this is the first reported evidence that polymorphisms involved in the NLR signalling pathway are associated with an increased risk of GC in a human population. Multivariate statistical analysis showed that the *CARD8*-rs11672725 TT genotype is a consistent risk factor for GC in Chinese individuals. *CARD8*, located at 19q13, encodes the caspase recruitment domain protein 8 (CARD8), also known as TUCAN, which has been reported to interact with NLRP3, suppress NF-κB activating signals and be overexpressed in human cancer tissues including ovarian, lung and breast cancer tissues [25–27]. To date, only one study, conducted in a German population, has assessed the role of this polymorphism in disease [28]. These authors addressed the association between *CARD8*-rs11672725 and colorectal cancer but failed to show any significant results (OR: 1.02, 95% CI: 0.86–1.21) [28]. Differences in cancer type and study population might account for these conflicting outcomes.

As *H. pylori* is known to be a major risk factor for GC, we also examined the effect of genetic polymorphisms involved in the NLR signalling pathway on *H. pylori* infection. On the multivariate analysis, *NLRP12*-rs2866112 consistently increased the risk of *H. pylori* infection in this ethnic Chinese population. Like NLRP3, NLRP12 has been shown to interact with ASC to generate an IL-1β-processing inflammasome [29]. To date few studies have addressed the role of NLRP12 in host resistance or susceptibility to infectious agents. Recently, however, a study by Vladimer et al. [30], demonstrated that NLRP12-deficient mice had higher mortality and increased bacterial loads after infection with *Yersinia pestis*, which correlated with decreased levels of IL-1β, IL18 and IFN-γ suggesting that NLRP12 might form an inflammasome in response to this bacteria. In contrast, Allen et al. [31] showed that NLRP12 did not significantly contribute to the *in vivo* host innate immune response to *Klebsiella pneumonia* or *Mycobacterium tuberculosis*. Further, Zaki et al. [32] have recently showed that NLRP12-deficient mice were highly resistant to *Salmonella enterica* serovar Typhimurium infection which was associated with the NLRP12-mediated inhibition of NF-κB and ERK activation. Thus, current evidence suggests that NLRP12 may play a dual pathogen-specific role, one in which a NLRP12 inflammasome-dependent response facilitates the eradication of the pathogen (e.g. *Y. pestis* infection), and a further one in which negative regulation of NF-κB signalling leads to survival and persistence of the pathogen (e.g. *S.* Typhimurium and possibly, *H. pylori* infection).

Given that GC is a complex disease, further multivariate statistical analyses were performed to investigate the potential interaction between *H. pylori* and the selected polymorphisms in the development of GC. Among *H. pylori*-infected individuals, those ones harbouring *CARD8*-rs10405717, *NLRP3*-rs12079994, *NLRP3*-rs3806265, *NLRP3*-rs4612666, *NLRP12*-rs2866112, *NLRP12*-rs4419163, *NLRX1*-rs10790286, *CASP1*-rs2282659, *CASP1*-rs530537 and *CASP1*-rs61751523, were at most risk of developing GC. Interestingly, a number of these genetic variants have previously been associated with diverse inflammation-related pathologies including cancer and autoimmune disorders [33,34].

Surprisingly, in the absence of *H. pylori* infection, *CARD8*-rs2043211 significantly decreased the risk of GC in these Chinese individuals. Consistent with this finding, a recent publication by Roberts et al. [35], found that the presence of the *CARD8*-rs2043211 T allele in combination with the *NLRP3*-rs35829419 C allele conferred a protective effect against Crohn's disease (CD) in Caucasian individuals (OR: 0.35, 95% CI: 0.15–0.82 and OR: 0.66, 95% CI: 0.48–0.90 for *CARD8* 1,1/*NLRP3* 1,2 and *CARD8* 1,2/*NLRP3* 1,1, respectively). In contrast, a study conducted in a Korean population revealed an association between *CARD8*-rs2043211 and ulcerative colitis (UC) (OR: 1.50, 95% CI: 1.12–2.00, *P*-value: 0.011) [36]. Further, *CARD8*-rs2043211 was also associated with elevated levels of IL-1β in female UC patients in this study [36]. Thus, further studies are required to elucidate the association between *CARD8*-rs2043211 and gastrointestinal inflammatory disorders among different ethnic groups.

Recently, inflammation has been considered the seventh hallmark of cancer and an enabling characteristic that facilitates the acquisition of the other hallmarks (tissue invasion/metastasis, limitless replicative potential, sustained angiogenesis, evasion of programmed-cell death (apoptosis), self-sufficiency in growth signals and insensitivity to growth-inhibitory signals) [37]. Inflammation initiated by innate immune cells, mainly macrophage subtypes, neutrophils, myeloid progenitors and mast cells [38–41], designed to fight infections and heal lesions, can instead result in unintentional support of multiple cancer hallmark functions, thereby manifesting the widely accepted tumour-promoting consequences of inflammatory responses [37]. Therefore, to assess the direct effect of *H. pylori* on the NLR signalling pathway, an important component of innate immunity that has been involved in gastric immunopathology, the expression of 84 genes was investigated in THP-1 cells challenged with two unrelated *H. pylori* strains (GC026 and 26695). Because pro-inflammatory cytokines play an essential role promoting inflammation in the context of gastrointestinal carcinogenesis [42], we first compared the expression of these genes between non-infected and *H. pylori*-infected THP-1 cells. Nine genes encoding pro-inflammatory cytokines (*IFNB1*, *IL1B*, *IL12B*, *IL6*, *IL33* and *TNF*) and

Table 3. Joint effect of *Helicobacter pylori* infection and genetic polymorphisms involved in the NOD-like receptors signalling pathway on gastric cancer risk.

Polymorphism	HP status	Genotype	Cases	Controls	OR	95% CI	*P*-value*
CARD8 rs10405717	HP (−)	CC	8	34	1		
	HP (−)	T carrier	6	45	0.57	0.18–1.79	0.3897
	HP (+)	CC	32	64	2.13	0.88–5.12	0.1051
	HP (+)	T carrier	40	69	**2.46**	**1.04–5.84**	**0.0504**
CARD8 rs2043211	HP (−)	AA	8	16	1		
	HP (−)	T carrier	6	63	**0.19**	**0.058–0.63**	**0.0071**
	HP (+)	AA	13	38	0.68	0.24–1.97	0.5833
	HP (+)	T carrier	59	95	1.24	0.50–3.08	0.8213
NLRP3 rs12079994	HP (−)	GG	10	59	1		
	HP (−)	A carrier	4	21	1.12	0.32–3.97	1
	HP (+)	GG	53	106	**2.95**	**1.40–6.23**	**0.0036**
	HP (+)	A carrier	19	27	**4.15**	**1.70–10.12**	**0.0019**
NLRP3 rs3806265	HP (−)	TT	4	23	1		
	HP (−)	C carrier	10	57	1.01	0.29–3.55	1
	HP (+)	TT	17	38	2.57	0.77–8.60	0.178
	HP (+)	C carrier	55	95	**3.33**	**1.09–10.13**	**0.0276**
NLRP3 rs4612666	HP (−)	CC	3	21	1		
	HP (−)	T carrier	11	59	1.31	0.33–5.14	1
	HP (+)	CC	18	41	3.07	0.81–11.63	0.1025
	HP (+)	T carrier	53	92	**4.03**	**1.15–14.16**	**0.0201**
NLRP12 rs2866112	HP (−)	CC	8	59	1		
	HP (−)	G carrier	6	22	2.01	0.63–6.46	0.34
	HP (+)	CC	29	69	**3.10**	**1.32–7.30**	**0.008**
	HP (+)	G carrier	43	67	**4.73**	**2.06–10.88**	**<0.0001**
NLRP12 rs4419163	HP (−)	TT	11	48	1		
	HP (−)	A carrier	3	31	0.42	0.11–1.64	0.243
	HP (+)	TT	35	67	**2.28**	**1.05–4.94**	**0.0459**
	HP (+)	A carrier	36	65	**2.42**	**1.12–5.23**	**0.0304**
NLRX1 rs10790286	HP (−)	TT	7	45	1		
	HP (−)	C carrier	7	36	1.25	0.40–3.89	0.7755
	HP (+)	TT	26	62	**2.70**	**1.08–6.76**	**0.0389**
	HP (+)	C carrier	46	74	**4.00**	**1.66–9.61**	**0.0011**
CASP1 rs2282659	HP (−)	AA	5	39	1		
	HP (−)	G carrier	9	42	1.67	0.52–5.42	0.563
	HP (+)	AA	38	78	**3.80**	**1.39–10.42**	**0.0087**

Table 3. Cont.

Polymorphism	HP status	Genotype	Cases	Controls	OR	95% CI	*P*-value*
	HP (+)	G carrier	34	57	**4.65**	**1.67–12.95**	**0.0021**
CASP1 rs530537	HP (−)	AA	5	39	1		
	HP (−)	G carrier	9	42	1.67	0.52–5.42	0.563
	HP (+)	AA	38	78	**3.80**	**1.39–10.42**	**0.0087**
	HP (+)	G carrier	34	57	**4.65**	**1.67–12.95**	**0.0021**
CASP1 rs61751523	HP (−)	TT	14	71	1		
	HP (−)	C carrier	0	11	0.21	0.01–3.85	0.358
	HP (+)	TT	62	126	**2.50**	**1.30–4.78**	**0.0054**
	HP (+)	C carrier	9	10	**4.56**	**1.57–13.28**	**0.0113**

HP, *Helicobacter pylori*; OR, odds ratio; CI, confidence intervals.
*Fisher's exact test two-tailed *P*-value.
doi:10.1371/journal.pone.0098899.t003

chemokines (*CXCL1*, *CXCL2, CCL5*) were found to be significantly up-regulated in THP-1 cells challenged with both *H. pylori* strains. These results are consistent with extensive evidence showing up-regulation of pro-inflammatory cytokines during *H. pylori* infection [43,44]. Interestingly, *CXCL1*, *CXCL2*, *CCL5*, *IL6*, *IL12B*, *TNF* and *IFNB1* were markedly up-regulated in THP-1 cells challenged with *H. pylori* GC026, a strain isolated from a GC patient and known to be positive for multiple virulence factors (*cagA*+, *cagE*+, *cagL*+, *cagT*+, *vacA* s1m1+, *babA*+, *oipA*+, *dupA*+ and *sabA*+), when compared to the reference strain *H. pylori* 26695. Further studies analysing the potential role of specific *H. pylori* virulence factors in the regulation of the NLR signalling pathway are required.

On the other hand, genes encoding a number of cytokines (*IL18*, *TNFSF11* and *CD40LG*) and chemokines (*CCL2* and *CCL7*) were found to be down-regulated in *H. pylori*-challenged THP-1 cells. Of these, only *CCL7* showed decreased expression in THP-1 cells challenged with both *H. pylori* GC026 and 26695 strains. Interestingly, *H. pylori*-induced down-regulation of chemokines and their receptors has been previously suggested as a novel mechanism of modulating neutrophil migration and activation in the gastric mucosa [45]. As *H. pylori* might exert a similar effect on *CCL7*, a chemokine that attracts monocytes and eosinophils and regulates macrophage function, this potential mechanism requires further investigation. Notably, we found expression of *IL18* to be significantly down-regulated only in the *H. pylori* GC026-challenged cells. Given that it has been reported that IL-18 counteracts the pro-inflammatory activities of IL-1β, thereby balancing control of *H. pylori* infection and prevention of excessive gastric immunopathology [10], it could be postulated that down-regulation of *IL18* contributes to the increased inflammation associated with GC.

The expression of thirteen genes encoding NLRs was analysed in the current study. Of these, five genes were significantly down-regulated at least two-fold in THP-1 cells challenged with *H. pylori* GC026 (*NLRC4*, *NLRC5*, *NLRP12* and *NLRX1*) and 26695 (*NLRC4*, *NLRC5* and *NLRP9*). Interestingly, *NLRP3,* a gene encoding a NLR that has previously been shown by Kim et al. [13] to cooperate with TLR2 and NOD2 for the secretion of IL-1β in *H. pylori*-infected DCs, was not differentially expressed in *H. pylori* infected-cells in our study. However, as described by others, there is considerable variation in phenotype, cytokine secretion, phagocytosis and T cell stimulating capabilities between monocytes, DCs and macrophages, in response to *H. pylori* infection [46]. Indeed, our results imply that other NLRs expressed in macrophages might be involved in *H. pylori* recognition and subsequent chronic inflammation including *NLRC4* (previously shown to be involved in the recognition of gram-negative bacteria type III and IV secretion systems [47,48]), *NLRC5* (a specific and master regulator of major histocompatibility complex (MHC) class I genes as well as related genes involved in MHC class I antigen presentation [49]) and *NLRP9* (recently associated with other inflammation-related disorders [50]).

In addition, our gene expression analyses showed *NLRP12* and *NLRX1*, two genes encoding molecules that have recently been suggested to negatively regulate canonical and non-canonical NF-κB signalling [32,51–54], to be markedly down-regulated in *H. pylori* GC026-challenged cells. Further, statistically significant up-regulation of *NFKB1* was only observed in *H. pylori* GC026-challenged cells. These results imply that, in THP-1 cells infected with highly virulent *H. pylori* strains, a dual mechanism may exacerbate the activity of the NF-κB signalling pathway, one of which increases host response to bacterial components leading to NF-κB activation through other PRRs, most likely TLRs, and

Table 4. Significant up-regulation of genes involved in the NOD-like receptor signalling pathway in a monocytic cell line upon exposure to *Helicobacter pylori*.

Regulation*	Gene symbol	Gene name	*Helicobacter pylori* GC026			*Helicobacter pylori* 26695		
			Median fold change	95% CI	*P*-value	Median fold change	95% CI	*P*-value
Up-regulated	*BIRC3*	Baculoviral IAP repeat containing 3	**12.29**	**(8.37, 16.20)**	**0.0016**	0.99	(0.00001, 2.11)	0.7263
	CASP5	Caspase 5, apoptosis-related cysteine peptidase	**3.09**	**(2.63, 3.55)**	**0.0004**	1.00	(1.00, 1.00)	0.0000
	CCL5	Chemokine (C-C motif) ligand 5	**2.28**	**(1.30, 3.26)**	**0.0366**	0.94	(0.00001, 2.19)	0.9940
	CXCL1	Chemokine (C-X-C motif) ligand 1	**22.45**	**(8.83, 36.08)**	**0.0006**	**2.95**	**(0.75, 5.14)**	**0.0188**
	CXCL2	Chemokine (C-X-C motif) ligand 2	**60.08**	**(27.53, 92.64)**	**0.0002**	20.73	(0.00001, 42.73)	0.0575
	IFNB1	Interferon, beta 1, fibroblast	**32.50**	**(15.41, 49.58)**	**0.0001**	3.49	(0.57, 6.41)	0.0642
	IL12A	Interleukin 12A	**3.59**	**(2.49, 4.69)**	**0.0003**	0.60	(0.00001, 1.32)	0.4203
	IL12B	Interleukin 12B	**434.20**	**(0.00001, 1093.32)**	**0.0399**	5.36	(0.00001, 17.63)	0.2003
	IL1B	Interleukin 1, beta	**3.70**	**(1.56, 5.83)**	**0.0086**	**63.26**	**(0.00001, 478.44)**	**0.0446**
	IL33	Interleukin 33	**4.78**	**(3.81, 5.74)**	**0.0000**	15.82	(0.00001, 43.71)	0.0522
	IL6	Interleukin 6	**262.39**	**(32.53, 492.24)**	**0.0008**	**94.57**	**(0.00001, 234.99)**	**0.0292**
	NFKB1	Nuclear factor of kappa light polypeptide gene enhancer in B-cells 1	**2.50**	**(1.90, 3.10)**	**0.0068**	0.58	(0.00001, 1.25)	0.4679
	NFKBIA	Nuclear factor of kappa light polypeptide gene enhancer in B-cells inhibitor, alpha	**6.60**	**(4.38, 8.82)**	**0.0001**	1.46	(0.43, 2.49)	0.3920
	P2RX7	Purinergic receptor P2X, ligand-gated ion channel, 7	**6.99**	**(5.17, 8.81)**	**0.0017**	3.10	(0.00001, 7.12)	0.2016
	PTGS2	Prostaglandin-endoperoxide synthase 2	**54.03**	**(10.88, 97.17)**	**0.0247**	**19.56**	**(7.32, 31.81)**	**0.0161**
	RIPK2	Receptor-interacting serine-threonine kinase 2	**3.09**	**(2.26, 3.92)**	**0.0035**	0.67	(0.13, 1.21)	0.4007
	TNF	Tumor necrosis factor	**32.12**	**(19.19, 45.05)**	**0.0003**	**14.32**	**(7.45, 21.19)**	**0.0108**

CI, confidence intervals. * Corresponds to genes showing at least two-fold changes (≥2 indicates up-regulation) and *P*-values<0.05, in THP-1 cells challenged with *H. pylori* GC026 and/or 26695.

doi:10.1371/journal.pone.0098899.t004

Table 5. Significant down-regulation of genes involved in the NOD-like receptor signalling pathway in a monocytic cell line upon exposure to *Helicobacter pylori*.

Regulation*	Gene symbol	Gene name	*Helicobacter pylori* GC026			*Helicobacter pylori* 26695		
			Median fold change	95% CI	*P*-value	Median fold change	95% CI	*P*-value
Down-regulated	*AIM2*	Absent in melanoma 2	**0.07**	**(0.00001, 0.15)**	**0.0016**	**0.06**	**(0.01, 0.11)**	**0.0110**
	BCL2L1	BCL2-like 1	**0.09**	**(0.02, 0.16)**	**0.0332**	0.23	(0.00001, 0.56)	0.1923
	CARD6	Caspase recruitment domain family, member 6	**0.29**	**(0.18, 0.41)**	**0.0029**	0.22	(0.00001, 0.59)	0.1449
	CASP1	Caspase 1, apoptosis-related cysteine peptidase	1.04	(0.85, 1.22)	0.7008	**0.32**	**(0.12, 0.52)**	**0.0182**
	CASP4	Caspase 4, apoptosis-related cysteine peptidase	0.54	(0.38, 0.71)	0.0093	**0.21**	**(0.11, 0.32)**	**0.0220**
	CASP8	Caspase 8, apoptosis-related cysteine peptidase	**0.28**	**(0.20, 0.36)**	**0.0054**	**0.06**	**(0.00001, 0.17)**	**0.0060**
	CCL2	Chemokine (C-C motif) ligand 2	1.40	(1.02, 1.79)	0.0737	**0.12**	**(0.05, 0.19)**	**0.0272**
	CCL7	Chemokine (C-C motif) ligand 7	0.59	(0.45, 0.73)	0.0124	**0.11**	**(0.00001, 0.24)**	**0.0417**
	CD40LG	CD40 ligand	**0.05**	**(0.04, 0.06)**	**0.0000**	2.15	(0.00001, 7.38)	0.3977
	CHUK	Conserved helix-loop-helix ubiquitous kinase	0.86	(0.32, 1.41)	0.4720	**0.20**	**(0.05, 0.35)**	**0.0223**
	CTSB	Cathepsin B	**0.44**	**(0.42, 0.46)**	**0.0000**	**0.15**	**(0.08, 0.22)**	**0.0013**
	FADD	Fas (TNFRSF6)-associated via death domain	**0.10**	**(0.00001, 0.20)**	**0.0255**	0.04	(0.00001, 0.15)	0.1890
	HSP90AA1	Heat shock protein 90 kDa alpha (cytosolic), class A member 1	0.63	(0.56, 0.71)	0.0031	**0.16**	**(0.10, 0.22)**	**0.0003**
	HSP90AB1	Heat shock protein 90 kDa alpha (cytosolic), class B member 1	0.96	(0.55, 1.38)	0.7407	**0.11**	**(0.01, 0.22)**	**0.0204**
	HSP90B1	Heat shock protein 90 kDa beta (Grp94), member 1	**0.42**	**(0.26, 0.58)**	**0.0040**	0.19	(0.05, 0.33)	0.0894
	IKBKB	Inhibitor of kappa light polypeptide gene enhancer in B-cells, kinase beta	0.65	(0.42, 0.87)	0.0970	**0.10**	**(0.03, 0.17)**	**0.0364**
	IL18	Interleukin 18	**0.43**	**(0.29, 0.57)**	**0.0072**	0.12	(0.02, 0.22)	0.0694
	IRF2	Interferon regulatory factor 2	**0.21**	**(0.13, 0.28)**	**0.0108**	0.09	(0.01, 0.17)	0.0796
	MAP3K7	Mitogen-activated protein kinase kinase kinase 7	0.54	(0.44, 0.64)	0.0071	**0.15**	**(0.07, 0.23)**	**0.0053**
	TAB1	TGF-beta activated kinase 1/MAP3K7 binding protein 1	**0.09**	**(0.05, 0.14)**	**0.0021**	0.09	(0.00001, 0.19)	0.1703
	TAB2	TGF-beta activated kinase 1/MAP3K7 binding protein 2	**0.39**	**(0.29, 0.50)**	**0.0093**	0.12	(0.00001, 0.27)	0.2276
	MAPK1	Mitogen-activated protein kinase 1	**0.18**	**(0.09, 0.26)**	**0.0047**	0.12	(0.03, 0.21)	0.0546
	MAPK11	Mitogen-activated protein kinase 11	0.91	(0.39, 1.43)	0.7202	**0.08**	**(0.00001, 0.17)**	**0.0459**

Table 5. Cont.

Regulation*	Gene symbol	Gene name	*Helicobacter pylori* GC026			*Helicobacter pylori* 26695		
			**Median fold change**	**95% CI**	***P*-value**	**Median fold change**	**95% CI**	***P*-value**
	MAPK12	Mitogen-activated protein kinase 12	**0.16**	**(0.09, 0.23)**	**0.0050**	0.12	(0.00001, 0.52)	0.2075
	MAPK13	Mitogen-activated protein kinase 13	0.34	(0.18, 0.51)	0.0560	**0.11**	**(0.01, 0.22)**	**0.0191**
	MAPK3	Mitogen-activated protein kinase 3	0.22	(0.05, 0.39)	0.0560	**0.05**	**(0.00001, 0.14)**	**0.0325**
	MAPK8	Mitogen-activated protein kinase 8	**0.40**	**(0.10, 0.70)**	**0.0318**	0.00	(0.00001, 0.03)	0.3739
	MAPK9	Mitogen-activated protein kinase 9	**0.30**	**(0.25, 0.35)**	**0.0002**	**0.14**	**(0.07, 0.21)**	**0.0141**
	MEFV	Mediterranean fever	**0.25**	**(0.15, 0.36)**	**0.0090**	0.17	(0.00001, 0.55)	0.1632
	MYD88	Myeloid differentiation primary response gene (88)	**0.11**	**(0.05, 0.17)**	**0.0096**	0.13	(0.00001, 0.29)	0.1700
	NLRC4	NLR family, CARD domain containing 4	**0.12**	**(0.07, 0.17)**	**0.0003**	**0.06**	**(0.01, 0.10)**	**0.0357**
	NLRC5	NLR family, CARD domain containing 5	**0.14**	**(0.10, 0.18)**	**0.0003**	**0.04**	**(0.02, 0.06)**	**0.0131**
	NLRP12	NLR family, pyrin domain containing 12	**0.03**	**(0.01, 0.04)**	**0.0163**	0.11	(0.00, 0.21)	0.1137
	NLRP9	NLR family, pyrin domain containing 9	0.77	(0.30, 1.24)	0.4548	**0.12**	**(0.00001, 0.26)**	**0.0005**
	NLRX1	NLR family member X1	**0.02**	**(0.00001, 0.05)**	**0.0176**	0.22	(0.00001, 0.85)	0.4216
	PEA15	Phosphoprotein enriched in astrocytes 15	0.49	(0.00001, 1.07)	0.2752	**0.27**	**(0.11, 0.42)**	**0.0112**
	PSTPIP1	Proline-serine-threonine phosphatase interacting protein 1	**0.13**	**(0.07, 0.19)**	**0.0066**	0.03	(0.00001, 0.06)	0.0541
	PYCARD	PYD and CARD domain containing	**0.05**	**(0.03, 0.07)**	**0.0001**	**0.03**	**(0.01, 0.05)**	**0.0068**
	RAGE	Renal tumor antigen	0.48	(0.00001, 0.99)	0.2113	**0.04**	**(0.00, 0.08)**	**0.0450**
	RELA	V-rel reticuloendotheliosis viral oncogene homolog A (avian)	**0.49**	**(0.32, 0.67)**	**0.0447**	0.26	(0.00001, 0.52)	0.1310
	SUGT1	SGT1, suppressor of G2 allele of SKP1 (S. cerevisiae)	0.52	(0.43, 0.60)	0.0006	**0.21**	**(0.01, 0.42)**	**0.0470**
	TNFSF11	Tumor necrosis factor (ligand) superfamily, member 11	**0.13**	**(0.00001, 0.26)**	**0.0092**	0.48	(0.00001, 1.69)	0.3440
	TXNIP	Thioredoxin interacting protein	**0.06**	**(0.02, 0.10)**	**0.0025**	0.06	(0.00001, 0.13)	0.1814
	XIAP	X-linked inhibitor of apoptosis	**0.42**	**(0.22, 0.62)**	**0.0429**	0.13	(0.00001, 0.27)	0.1044

CI, confidence intervals.
*Corresponds to genes showing at least two-fold changes (≤0.5 indicates down-regulation) and *P*-values<0.05, in THP-1 cells challenged with *H. pylori* GC026 and/or 26695.
doi:10.1371/journal.pone.0098899.t005

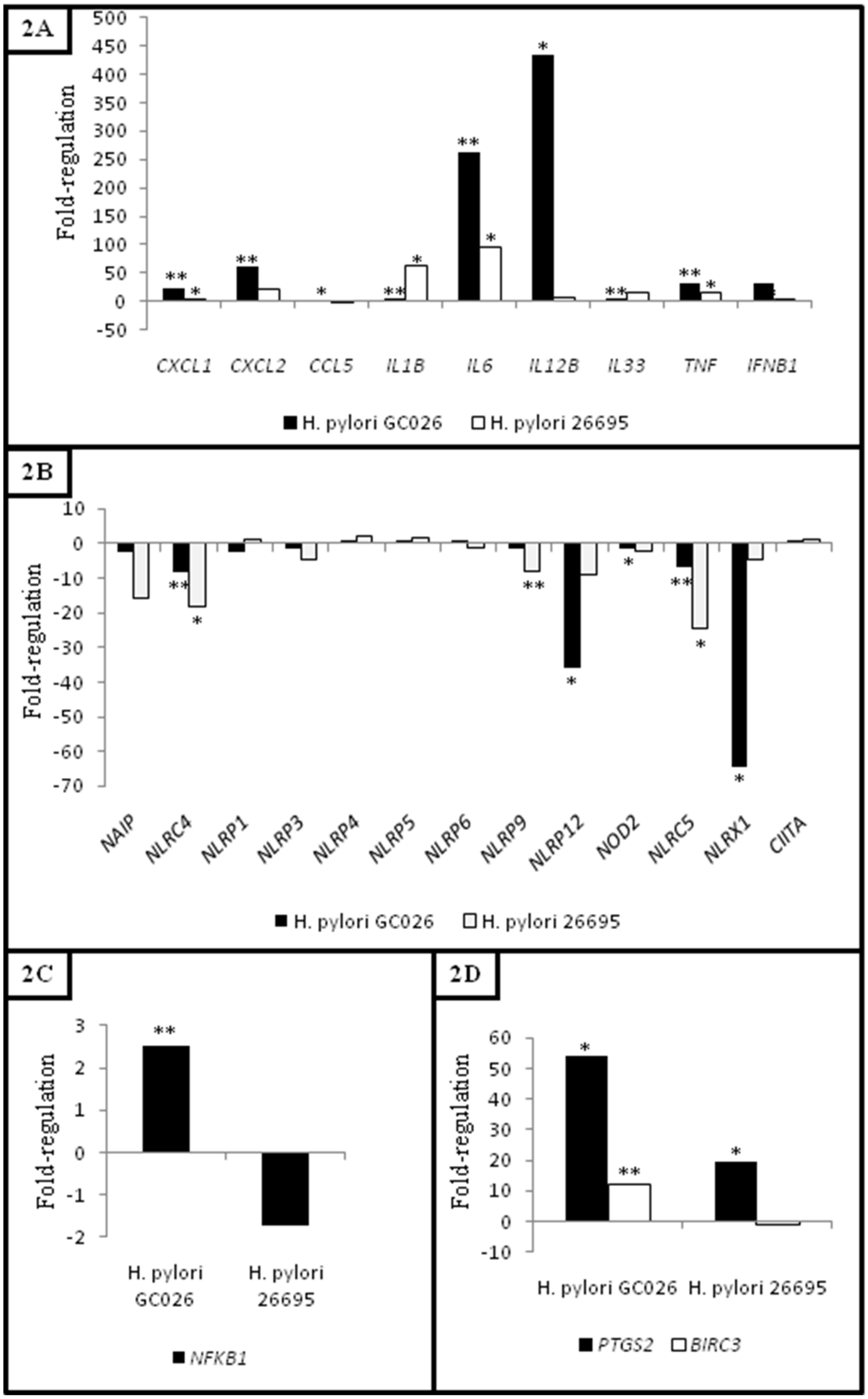

Figure 1. *Helicobacter pylori* up-regulates *NFKB1* and NF-κB target genes, and down-regulates NF-κB-negative regulators. A) Gene expression of cytokines and chemokines in *H. pylori*-challenged THP-1 cells, **B)** Gene expression of NOD-like receptors in *H. pylori*-challenged THP-1 cells, **C)** *NFKB1* expression in *H. pylori*-challenged THP-1 cells and **D)** *PTGS2* and *BIRC3* expression in *H. pylori*-challenged THP-1 cells. Fold-change (2^(−Delta Delta Ct)) is the normalized gene expression (2^(−Delta Ct)) in THP-1 cells challenged with *H. pylori* (GC026 and 26695) divided the normalized gene expression (2^(−Delta Ct)) in their respective control group. Fold-regulation represents fold-change results in a biologically meaningful way. Fold-change values greater than one indicate up-regulation, and the fold-regulation is equal to the fold-change. Fold-change values less than one indicate down-regulation, and the fold-regulation is the negative inverse of the fold-change. Fold-difference compared to the control group showing a **P*-value<0.05 and a ***P*-value<0.01. *P*-values were obtained with a Student's t-test.
doi:10.1371/journal.pone.0098899.g001

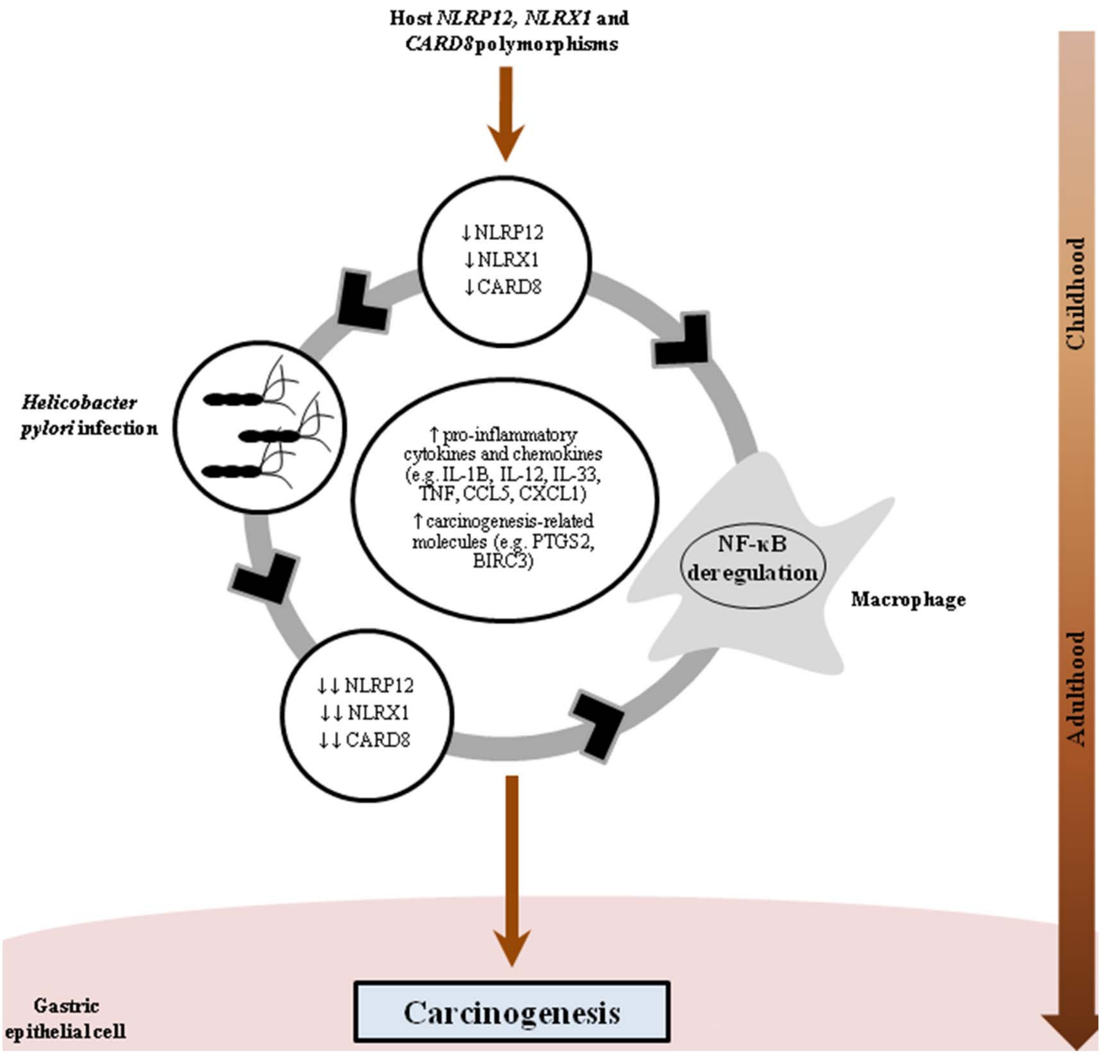

Figure 2. Polymorphisms in the NOD-like receptor signalling pathway increase the risk of *Helicobacter pylori*-related gastric cancer. Individuals harbouring *NLRP12*, *NLRX1* and *CARD8* polymorphisms would have decreased levels or defective NLRP12, NLRX1 and CARD8, and thus, would be more susceptible to acquisition of *H. pylori* in childhood and present deregulation of NF-κB. Once established, *H. pylori* infection appears to intensify the decreased expression of these NF-κB-negative regulators. These factors would lead to the production of pro-inflammatory cytokines (*IFNB1*, *IL1*, *IL-12B*, *IL6*, *IL33* and *TNF*), chemokines (*CXCL1*, *CXCL2*, *CCL5*) and carcinogenesis-related molecules (*PTGS2* and *BIRC3*), among others, which would facilitate the sequence of events that characterises GC development including inflammation, atrophy, intestinal metaplasia, dysplasia, carcinoma *in situ*, and finally invasive gastric cancer.
doi:10.1371/journal.pone.0098899.g002

secondly, down-regulation of the NF-κB-negative regulators NLRP12 and NLRX1.

NLRP12-mediated attenuation of the non-canonical NF-κB pathway has been suggested to be due to NLRP12's ability to associate with the NF-κB inducing kinase (NIK), which subsequently induces proteasome-dependent degradation of this protein [52]. In addition, evidence suggests that NLRP12 regulates the canonical NF-κB signalling pathway by targeting the interleukin-1 receptor-associated kinase 1 (IRAK1), which results in blockage of IRAK-1 hyperphosphorylation [53]. Similarly, NLRX1 was found to target TNF receptor-associated factor 6 (TRAF6) inhibiting its capacity to signal to NF-κB [51]. Further, upon LPS stimulation, NLRX1 is rapidly ubiquitinated, disassociates from TRAF6, and then binds to the I kappa B kinase (IKK) complex, resulting in inhibition of IKKα and IKKβ phosphorylation and NF-κB activation [54]. In the context of gastrointestinal carcinogenesis, Zaki et al. [55] have demonstrated that NLRP12 plays an essential role in the suppression of pro-inflammatory cytokines and chemokines by controlling the activation of NF-κB and extracellular-signal-regulated kinase (ERK) pathways in response to microbial components, colon inflammation and colorectal tumorigenesis. Further, Allen et al. [56] have found that NLRP12-deficient mice were highly susceptible to colitis and colitis-associated colon cancer showing elevated non-canonical NF-κB activation and increased expression of target genes associated with cancer, including *CXCL13* and *CXCL12*.

PTGS2 and *BIRC3*, two molecules known to be involved in carcinogenesis, were significantly up-regulated in *H. pylori*-GC026-challenged cells. Prostaglandin-endoperoxide synthase 2 (PTGS2), which is also termed cyclooxygenase 2 (COX2), is the key enzyme that catalyses the conversion of arachidonic acid to prostaglandins. Extensive evidence supports a pivotal role of PTGS2 in gastric inflammation and carcinogenesis [57]. In addition, Zaki et al. [58] showed that elevated PTGS2 expression was evident in colonic

tumours of NLRP12-deficient mice leading to the possibility that, in the current study, decreased expression of *NLRP12* is related to increased *PTGS2* levels in *H. pylori* GC026-challenged cells.

Further, because baculoviral IAP repeat containing 3 (BIRC3), also known as cellular inhibitor of apoptosis protein 2 (cIAP2), is a member of the inhibitor of apoptosis protein (IAP) family, and is over-expressed in most cancer tissues [59], it can be postulated that stimulus with *H. pylori* GC026 elevates the already high basal levels of *BIRC3* in THP-1 cells, a cell line derived from an acute monocytic leukemia patient. Consistent with this view, Maeda et al. [60] have shown that *H. pylori* infection increases BIRC3 expression in human GC and cervical cancer cell lines. Further, *H. pylori* has been shown to increase the expression of BIRC3 in infected-mice after 2 weeks of exposure [61]. In addition, as knocking down of BIRC3 resulted in a 30% decrease in cell proliferation, a 20% increase in apoptosis and delayed migration of SGC-7901 cells, it has been postulated that BIRC3 is a potential target for GC therapy [61].

In conclusion, the association between *CARD8*-rs11672725 and GC was a consistent and novel finding in this study. Further, *NLRP12*-rs2866112 was found to increase the risk of *H. pylori* infection in this population, the main risk factor for GC. Additional analyses in our ethnic Chinese population showed that the concomitant presence of polymorphisms involved in the NLR signalling pathway and *H. pylori* infection dramatically increased the risk of GC in Chinese. Our gene expression analyses showed five genes encoding NLRs to be significantly regulated in *H. pylori*-challenged cells (*NLRC4*, *NLRC5*, *NLRP9*, *NLRP12* and *NLRX1*). Interestingly, *NLRP12* and *NLRX1*, two known NF-κB negative regulators, were markedly down-regulated, and *NFKB1* as well as several NF-κB target genes encoding pro-inflammatory cytokines (*IFNB1*, *IL12B*, *IL6* and *TNF*), chemokines (*CXCL1*, *CXCL2*, *CCL5)* and molecules involved in carcinogenesis (*PTGS2* and *BIRC3*) were markedly up-regulated, in THP-1 cells infected with a highly virulent *H. pylori* strain isolated from a GC patient. Based on our findings, we propose a synergistic interaction between NLRs and *H. pylori* in GC pathogenesis, which over time, could facilitate the sequence of events that characterises GC development including inflammation, atrophy, metaplasia, dysplasia, carcinoma *in situ*, and finally invasive GC (Figure 2).

Supporting Information

Figures S1 This file contains Figures S1 and S2. *Helicobacter pylori* influences the expression of several molecules involved in the NOD-like receptor signalling pathway. THP-1 cells were challenged with two *H. pylori* strains (GC026 and 26695). Total RNA was extracted from cells after 6 hours infection. Gene expression was detected by quantitative RT-PCR in triplicates. **S1)** Gene expression of 84 molecules involved in the NOD-like receptor (NLR) signalling pathway in *H. pylori* GC026-challenged THP-1 cells. **S2)** Gene expression of 84 molecules involved in the NLR signalling pathway in *H. pylori* 26695-challenged THP-1 cells. The x-axis plots the log2 of the fold-differences, while the y-axis plots their p-values based on a student's t-test of the replicate raw Ct data. The red and green circles outside the two vertical lines indicate fold-differences >2. Circles in the volcano plot above the blue line identify fold-differences showing *P*-values<0.05.
(TIF)

Figure S3 *Helicobacter pylori* down-regulates NLRX1 levels in THP-1 cells. The levels of NLRX1 were significantly decreased in THP-1 cells challenged with either *H. pylori* strain GC26 or 26695. A representative experiment of the triplicates performed is shown.
(TIF)

Tables S1. This contains files Tables S1–S7. Table S1. Genetic polymorphisms in genes involved in the NOD-like receptors signalling pathway included in the current study. **Table S2.** PCR primer sequences and thermal conditions used for genotyping of *NLRP3* and *CARD8* polymorphisms in gastric cancer patients and functional dyspepsia controls. **Table S3.** Clinical characteristics of gastric cancer patients and functional dyspepsia controls. **Table S4.** Association between the *NLRP3* 42 bp-VNTR polymorphism and risk of gastric cancer in ethnic Chinese individuals (bivariate statistical analysis). **Table S5.** Association between *CARD8-NLRP12*, *NLRP3*, *NLRX1-CASP1* and *ASC* haplotypes and gastric cancer in an ethnic Chinese population. **Table S6.** Association between polymorphisms involved in the NOD-like receptors signalling pathway and risk of *Helicobacter pylori* infection in ethnic Chinese individuals (bivariate statistical analysis). **Table S7.** Effect of *Helicobacter pylori* infection on the expression of genes involved in the NOD-like receptors signalling pathway.
(DOC)

Acknowledgments

The authors would like to thank all individuals with GC and FD who participated in the ethnic Chinese case-control study.

Author Contributions

Conceived and designed the experiments: NCR NOK KLG KMF HMM. Performed the experiments: NCR NOK. Analyzed the data: NCR NOK HMM. Contributed reagents/materials/analysis tools: NCR NOK KLG KMF HMM. Wrote the paper: NCR NOK KLG KMF HMM.

References

1. Ferlay J SI, Ervik M, Dikshit R, Eser S, Mathers C, et al. (2013) GLOBOCAN 2012 v1.0, Cancer Incidence and Mortality Worldwide. IARC CancerBase No 11 [Internet]. Lyon, France: International Agency for Research on Cancer.
2. Parkin DM, Bray F, Ferlay J, Pisani P (2005) Global cancer statistics, 2002. CA Cancer J Clin 55: 74–108.
3. Gonzalez CA, Sala N, Capella G (2002) Genetic susceptibility and gastric cancer risk. Int J Cancer 100: 249–260.
4. Macarthur M, Hold GL, El-Omar EM (2004) Inflammation and Cancer II. Role of chronic inflammation and cytokine gene polymorphisms in the pathogenesis of gastrointestinal malignancy. Am J Physiol Gastrointest Liver Physiol 286: G515–520.
5. Davis BK, Wen H, Ting JP (2011) The inflammasome NLRs in immunity, inflammation, and associated diseases. Annu Rev Immunol 29: 707–735.
6. Unterholzner L, Keating SE, Baran M, Horan KA, Jensen SB, et al. (2010) IFI16 is an innate immune sensor for intracellular DNA. Nat Immunol 11: 997–1004.
7. Schroder K, Tschopp J (2010) The inflammasomes. Cell 140: 821–832.
8. Martinon F, Mayor A, Tschopp J (2009) The inflammasomes: guardians of the body. Annu Rev Immunol 27: 229–265.
9. Basak C, Pathak SK, Bhattacharyya A, Mandal D, Pathak S, et al. (2005) NF-kappaB- and C/EBPbeta-driven interleukin-1beta gene expression and PAK1-mediated caspase-1 activation play essential roles in interleukin-1beta release from Helicobacter pylori lipopolysaccharide-stimulated macrophages. J Biol Chem 280: 4279–4288.
10. Hitzler I, Sayi A, Kohler E, Engler DB, Koch KN, et al. (2012) Caspase-1 has both proinflammatory and regulatory properties in Helicobacter infections, which are differentially mediated by its substrates IL-1beta and IL-18. J Immunol 188: 3594–3602.
11. Shimada M, Ando T, Peek RM, Watanabe O, Ishiguro K, et al. (2008) Helicobacter pylori infection upregulates interleukin-18 production from gastric epithelial cells. Eur J Gastroenterol Hepatol 20: 1144–1150.
12. Yang JC, Yang HC, Shun CT, Wang TH, Chien CT, et al. (2013) Catechins and Sialic Acid Attenuate Helicobacter pylori-Triggered Epithelial Caspase-1

Activity and Eradicate Helicobacter pylori Infection. Evid Based Complement Alternat Med 2013: 248585.
13. Kim DJ, Park JH, Franchi L, Backert S, Nunez G (2013) The Cag pathogenicity island and interaction between TLR2/NOD2 and NLRP3 regulate IL-1beta production in Helicobacter pylori infected dendritic cells. Eur J Immunol 43: 2650–8.
14. Talley NJ, Stanghellini V, Heading RC, Koch KL, Malagelada JR, et al. (1999) Functional gastroduodenal disorders. Gut 45 Suppl 2: II37–42.
15. Mitchell H, English DR, Elliott F, Gengos M, Barrett JH, et al. (2008) Immunoblotting using multiple antigens is essential to demonstrate the true risk of Helicobacter pylori infection for gastric cancer. Aliment Pharmacol Ther 28: 903–910.
16. Jurinke C, van den Boom D, Cantor CR, Koster H (2002) The use of MassARRAY technology for high throughput genotyping. Adv Biochem Eng Biotechnol 77: 57–74.
17. Oeth P BM, Park C, Kosman D, del Mistro G, van den Boom D, Jurinke C (2005) iPLEX Assay: Increased Plexing Efficiency and Flexibility for MassARRAY System Through Single Base Primer Extension with Mass-Modified Terminators. Sequenom. pp. 12.
18. Slatkin M, Excoffier L (1996) Testing for linkage disequilibrium in genotypic data using the Expectation-Maximization algorithm. Heredity 76 (Pt 4): 377–383.
19. Excoffier L, Slatkin M (1995) Maximum-likelihood estimation of molecular haplotype frequencies in a diploid population. Mol Biol Evol 12: 921–927.
20. Excoffier L, Laval G, Schneider S (2005) Arlequin (version 3.0): an integrated software package for population genetics data analysis. Evol Bioinform Online 1: 47–50.
21. Gunaletchumy SP, Teh X, Khosravi Y, Ramli NS, Chua EG, et al. (2012) Draft genome sequences of Helicobacter pylori isolates from Malaysia, cultured from patients with functional dyspepsia and gastric cancer. J Bacteriol 194: 5695–5696.
22. Schmidt HM, Andres S, Nilsson C, Kovach Z, Kaakoush NO, et al. (2010) The cag PAI is intact and functional but HP0521 varies significantly in Helicobacter pylori isolates from Malaysia and Singapore. Eur J Clin Microbiol Infect Dis 29: 439–451.
23. Akopyants NS, Eaton KA, Berg DE (1995) Adaptive mutation and cocolonization during Helicobacter pylori infection of gnotobiotic piglets. Infect Immun 63: 116–121.
24. Tsuchiya S, Kobayashi Y, Goto Y, Okumura H, Nakae S, et al. (1982) Induction of maturation in cultured human monocytic leukemia cells by a phorbol diester. Cancer Res 42: 1530–1536.
25. Agostini L, Martinon F, Burns K, McDermott MF, Hawkins PN, et al. (2004) NALP3 forms an IL-1beta-processing inflammasome with increased activity in Muckle-Wells autoinflammatory disorder. Immunity 20: 319–325.
26. Bouchier-Hayes L, Conroy H, Egan H, Adrain C, Creagh EM, et al. (2001) CARDINAL, a novel caspase recruitment domain protein, is an inhibitor of multiple NF-kappa B activation pathways. J Biol Chem 276: 44069–44077.
27. Pathan N, Marusawa H, Krajewska M, Matsuzawa S, Kim H, et al. (2001) TUCAN, an antiapoptotic caspase-associated recruitment domain family protein overexpressed in cancer. J Biol Chem 276: 32220–32229.
28. Mockelmann N, von Schonfels W, Buch S, von Kampen O, Sipos B, et al. (2009) Investigation of innate immunity genes CARD4, CARD8 and CARD15 as germline susceptibility factors for colorectal cancer. BMC Gastroenterol 9: 79.
29. Wang L, Manji GA, Grenier JM, Al-Garawi A, Merriam S, et al. (2002) PYPAF7, a novel PYRIN-containing Apaf1-like protein that regulates activation of NF-kappa B and caspase-1-dependent cytokine processing. J Biol Chem 277: 29874–29880.
30. Vladimer GI, Weng D, Paquette SW, Vanaja SK, Rathinam VA, et al. (2012) The NLRP12 inflammasome recognizes Yersinia pestis. Immunity 37: 96–107.
31. Allen IC, McElvania-TeKippe E, Wilson JE, Lich JD, Arthur JC, et al. (2013) Characterization of NLRP12 during the in vivo host immune response to Klebsiella pneumoniae and Mycobacterium tuberculosis. PLoS One 8: e60842.
32. Zaki MH, Man SM, Vogel P, Lamkanfi M, Kanneganti TD (2014) Salmonella exploits NLRP12-dependent innate immune signaling to suppress host defenses during infection. Proc Natl Acad Sci U S A 111: 385–390.
33. Lavender NA, Rogers EN, Yeyeodu S, Rudd J, Hu T, et al. (2012) Interaction among apoptosis-associated sequence variants and joint effects on aggressive prostate cancer. BMC Med Genomics 5: 11.
34. Day TG, Ramanan AV, Hinks A, Lamb R, Packham J, et al. (2008) Autoinflammatory genes and susceptibility to psoriatic juvenile idiopathic arthritis. Arthritis Rheum 58: 2142–2146.
35. Roberts RL, Topless RK, Phipps-Green AJ, Gearry RB, Barclay ML, et al. (2010) Evidence of interaction of CARD8 rs2043211 with NALP3 rs35829419 in Crohn's disease. Genes Immun 11: 351–356.
36. Yang SK, Kim H, Hong M, Lim J, Choi E, et al. (2011) Association of CARD8 with inflammatory bowel disease in Koreans. J Hum Genet 56: 217–223.
37. Hanahan D, Weinberg RA (2011) Hallmarks of cancer: the next generation. Cell 144: 646–674.
38. Coffelt SB, Lewis CE, Naldini L, Brown JM, Ferrara N, et al. (2010) Elusive identities and overlapping phenotypes of proangiogenic myeloid cells in tumors. Am J Pathol 176: 1564–1576.
39. De Palma M, Murdoch C, Venneri MA, Naldini L, Lewis CE (2007) Tie2-expressing monocytes: regulation of tumor angiogenesis and therapeutic implications. Trends Immunol 28: 519–524.
40. DeNardo DG, Andreu P, Coussens LM (2010) Interactions between lymphocytes and myeloid cells regulate pro- versus anti-tumor immunity. Cancer Metastasis Rev 29: 309–316.
41. Johansson M, Denardo DG, Coussens LM (2008) Polarized immune responses differentially regulate cancer development. Immunol Rev 222: 145–154.
42. Karin M, Greten FR (2005) NF-kappaB: linking inflammation and immunity to cancer development and progression. Nat Rev Immunol 5: 749–759.
43. Yamaoka Y, Kita M, Kodama T, Sawai N, Kashima K, et al. (1997) Induction of various cytokines and development of severe mucosal inflammation by cagA gene positive Helicobacter pylori strains. Gut 41: 442–451.
44. Wen S, Felley CP, Bouzourene H, Reimers M, Michetti P, et al. (2004) Inflammatory gene profiles in gastric mucosa during Helicobacter pylori infection in humans. J Immunol 172: 2595–2606.
45. Schmausser B, Josenhans C, Endrich S, Suerbaum S, Sitaru C, et al. (2004) Downregulation of CXCR1 and CXCR2 expression on human neutrophils by Helicobacter pylori: a new pathomechanism in H. pylori infection? Infect Immun 72: 6773–6779.
46. Fehlings M, Drobbe L, Moos V, Renner Viveros P, Hagen J, et al. (2012) Comparative analysis of the interaction of Helicobacter pylori with human dendritic cells, macrophages, and monocytes. Infect Immun 80: 2724–2734.
47. Faure E, Mear JB, Faure K, Normand S, Couturier-Maillard A, et al. (2014) Pseudomonas aeruginosa Type-3 secretion system dampens host defense by exploiting the NLRC4-coupled inflammasome. Am J Respir Crit Care Med.
48. Silveira TN, Zamboni DS (2010) Pore formation triggered by Legionella spp. is an Nlrc4 inflammasome-dependent host cell response that precedes pyroptosis. Infect Immun 78: 1403–1413.
49. Yao Y, Wang Y, Chen F, Huang Y, Zhu S, et al. (2012) NLRC5 regulates MHC class I antigen presentation in host defense against intracellular pathogens. Cell Res 22: 836–847.
50. Tadaki H, Saitsu H, Nishimura-Tadaki A, Imagawa T, Kikuchi M, et al. (2011) De novo 19q13.42 duplications involving NLRP gene cluster in a patient with systemic-onset juvenile idiopathic arthritis. J Hum Genet 56: 343–347.
51. Allen IC, Moore CB, Schneider M, Lei Y, Davis BK, et al. (2011) NLRX1 protein attenuates inflammatory responses to infection by interfering with the RIG-I-MAVS and TRAF6-NF-kappaB signaling pathways. Immunity 34: 854–865.
52. Lich JD, Williams KL, Moore CB, Arthur JC, Davis BK, et al. (2007) Monarch-1 suppresses non-canonical NF-kappaB activation and p52-dependent chemokine expression in monocytes. J Immunol 178: 1256–1260.
53. Williams KL, Lich JD, Duncan JA, Reed W, Rallabhandi P, et al. (2005) The CATERPILLER protein monarch-1 is an antagonist of toll-like receptor-, tumor necrosis factor alpha-, and Mycobacterium tuberculosis-induced pro-inflammatory signals. J Biol Chem 280: 39914–39924.
54. Xia X, Cui J, Wang HY, Zhu L, Matsueda S, et al. (2011) NLRX1 negatively regulates TLR-induced NF-kappaB signaling by targeting TRAF6 and IKK. Immunity 34: 843–853.
55. Zaki MH, Boyd KL, Vogel P, Kastan MB, Lamkanfi M, et al. (2010) The NLRP3 inflammasome protects against loss of epithelial integrity and mortality during experimental colitis. Immunity 32: 379–391.
56. Allen IC, Wilson JE, Schneider M, Lich JD, Roberts RA, et al. (2012) NLRP12 suppresses colon inflammation and tumorigenesis through the negative regulation of noncanonical NF-kappaB signaling. Immunity 36: 742–754.
57. Wu WK, Sung JJ, Lee CW, Yu J, Cho CH (2010) Cyclooxygenase-2 in tumorigenesis of gastrointestinal cancers: an update on the molecular mechanisms. Cancer Lett 295: 7–16.
58. Zaki MH, Vogel P, Malireddi RK, Body-Malapel M, Anand PK, et al. (2011) The NOD-like receptor NLRP12 attenuates colon inflammation and tumorigenesis. Cancer Cell 20: 649–660.
59. Tamm I, Kornblau SM, Segall H, Krajewski S, Welsh K, et al. (2000) Expression and prognostic significance of IAP-family genes in human cancers and myeloid leukemias. Clin Cancer Res 6: 1796–1803.
60. Maeda S, Yoshida H, Mitsuno Y, Hirata Y, Ogura K, et al. (2002) Analysis of apoptotic and antiapoptotic signalling pathways induced by Helicobacter pylori. Gut 50: 771–778.
61. Li Z, Chen J, Chan KW, Qiao L, Wong BC (2011) A possible role of cIAP2 in Helicobacter pylori-associated gastric cancer. Cancer Lett 313: 192–200.
